# The ETS-family transcription factor PU.1 is a critical regulator of the inhibitory Fcγ receptor IIB expression in humans

**DOI:** 10.1093/jimmun/vkaf109

**Published:** 2025-05-26

**Authors:** Matthew J Carter, Yury D Bogdanov, Rosanna C Smith, Kerry L Cox, Sarah Frampton, Lili Ferson, Russel B Foxall, Khiyam Hussain, Jonathan C Strefford, Stephen A Beers, Mark S Cragg

**Affiliations:** Antibody & Vaccine Group, Centre for Cancer Immunology, School of Cancer Sciences, Faculty of Medicine, Southampton General Hospital, University of Southampton, Southampton, United Kingdom; Antibody & Vaccine Group, Centre for Cancer Immunology, School of Cancer Sciences, Faculty of Medicine, Southampton General Hospital, University of Southampton, Southampton, United Kingdom; Antibody & Vaccine Group, Centre for Cancer Immunology, School of Cancer Sciences, Faculty of Medicine, Southampton General Hospital, University of Southampton, Southampton, United Kingdom; Antibody & Vaccine Group, Centre for Cancer Immunology, School of Cancer Sciences, Faculty of Medicine, Southampton General Hospital, University of Southampton, Southampton, United Kingdom; Antibody & Vaccine Group, Centre for Cancer Immunology, School of Cancer Sciences, Faculty of Medicine, Southampton General Hospital, University of Southampton, Southampton, United Kingdom; Cancer Genomics Group, School of Cancer Sciences, Faculty of Medicine, Southampton General Hospital, University of Southampton, Southampton, United Kingdom; Antibody & Vaccine Group, Centre for Cancer Immunology, School of Cancer Sciences, Faculty of Medicine, Southampton General Hospital, University of Southampton, Southampton, United Kingdom; Cancer Genomics Group, School of Cancer Sciences, Faculty of Medicine, Southampton General Hospital, University of Southampton, Southampton, United Kingdom; Antibody & Vaccine Group, Centre for Cancer Immunology, School of Cancer Sciences, Faculty of Medicine, Southampton General Hospital, University of Southampton, Southampton, United Kingdom; Antibody & Vaccine Group, Centre for Cancer Immunology, School of Cancer Sciences, Faculty of Medicine, Southampton General Hospital, University of Southampton, Southampton, United Kingdom; Oxford Vaccine Group, Centre for Clinical Vaccinology and Tropical Medicine, Churchill Hospital, Headington, Oxford, United Kingdom; Antibody & Vaccine Group, Centre for Cancer Immunology, School of Cancer Sciences, Faculty of Medicine, Southampton General Hospital, University of Southampton, Southampton, United Kingdom; Cancer Genomics Group, School of Cancer Sciences, Faculty of Medicine, Southampton General Hospital, University of Southampton, Southampton, United Kingdom; Antibody & Vaccine Group, Centre for Cancer Immunology, School of Cancer Sciences, Faculty of Medicine, Southampton General Hospital, University of Southampton, Southampton, United Kingdom; Institute for Life Sciences, University of Southampton, Southampton, United Kingdom; Antibody & Vaccine Group, Centre for Cancer Immunology, School of Cancer Sciences, Faculty of Medicine, Southampton General Hospital, University of Southampton, Southampton, United Kingdom; Institute for Life Sciences, University of Southampton, Southampton, United Kingdom

**Keywords:** Fcγ receptors, FCGR2B, gene regulation, immunotherapy, PU.1

## Abstract

The inhibitory Fc gamma receptor IIB (FcγRIIB) is a critical determinant of humoral immunity. By providing feedback inhibition, through inhibitory signalling or competition for antibody Fc engagement, it counterbalances and contextualises cellular responses to signals emanating from co-ligated activating receptors, such as the B-cell receptor and activating FcγR. These activities collectively suppress the emergence of B- cell-mediated autoimmune disease and immune complex-mediated pathologies. However, FcγRIIB upregulation within the tumour microenvironment limits the efficacy of monoclonal antibody (mAb)-mediated immunotherapy of cancer.

While the functional significance of FcγRIIB is well established in mice, its physiological roles and the regulatory mechanisms governing its expression remain incompletely understood in humans. Here we characterise the molecular determinants of FcγRIIB expression in human immune models and primary cells. Our findings reveal that the ETS-family transcription factor PU.1 plays a crucial role in regulating basal and inducible FcγRIIB expression. Moreover, when co-expressed, PU.1 co-operates with the related ETS-family member SPIB to drive FcγRIIB expression. PU.1 binding to the proximal FcγRIIB promoter elicits transcription, at least in part, through recruitment of the CBP/p300 transcriptional co-activators. Interestingly, similar mechanisms are also observed at the proximal promoters of the activating FcγRI and FcγRIIA, suggesting that additional, potentially lineage specific, factors cooperate with PU.1 to drive the distinct expression patterns of these FcγR. These insights pave the way for future investigations aimed at understanding the molecular mechanisms responsible for cell lineage-specific FcγR expression and subsequently manipulating them for therapeutic purposes.

## Graphical Abstract

**Figure:**
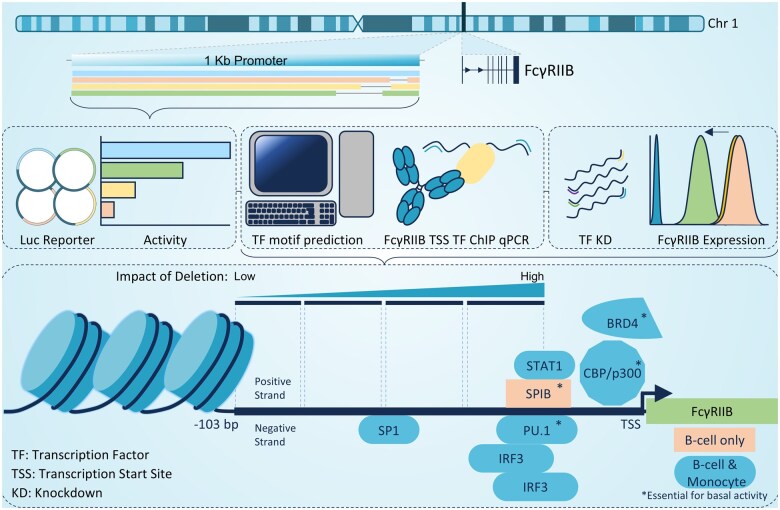

## Introduction

Functional insights from murine models have revealed Fcγ receptor (FcγR) IIB (FcγRIIB) as a critical determinant of immune homeostasis and modulator of responses to therapeutic monoclonal antibodies (mAbs).^1–3^ In the mouse, FcγRIIB is widely expressed as 1 of 2 cell surface isoforms (FcγRIIB1 or FcγRIIB2) in both hematopoietic (including monocytes, B-lymphocytes, eosinophils, basophils, and macrophages^4–6^) and select non-hematopoietic cell types.^7^^,^^8^ While FcγRIIB1 is principally expressed by B-cells, FcγRIIB2 represents the most dominant isoform in cells of the myeloid lineage.^1^^,^^3^ In this system, FcγRIIB counterbalances and contextualizes signals emanating from co-ligated activating receptors, such as the B-cell receptor (BCR) and activating FcγR.^9–12^ Through immunoreceptor tyrosine-based inhibitory motif (ITIM)-dependent gating of BCR signals,^11^ FcγRIIB increases cellular activation thresholds^9^^,^^11^ and contributes to B-cell tolerance mechanisms.^10^^,^^13^^,^^14^ FcγRIIB is particularly required for germinal center (GC) tolerance,^10^^,^^13–16^ where upregulation of its expression prevents the emergence of high affinity autoantibody responses.^16^^,^^17^

In addition to these B-cell-intrinsic effects,^15^^,^^17^ FcγRIIB restrains activating FcγR-dependent immunological processes.^12^^,^^18–20^ Accordingly, dysregulated FcγRIIB expression or function is widely implicated in the development of immune complex (IC)-mediated autoimmune disease^10^^,^^13^^,^^15–17^ and in resistance to direct-targeting mAb therapies in mice.^21–23^ Direct-targeting mAbs (eg, rituximab) harness activating FcγRs to delete opsonized cells via antibody-dependent cellular cytotoxicity (ADCC) and/or antibody-dependent cellular phagocytosis (ADCP).^24^^,^^25^ Of these, mononuclear phagocyte-mediated ADCP appears the most prominent in vivo effector mechanism in mice.^19^^,^^26^^,^^27^ FcγRIIB limits target cell depletion^20^^,^^21^ through competition with activating FcγRs for therapeutic antibody Fc engagement^28^ and via ITIM-mediated inhibition of activating FcγR signaling.^29^ In addition to these effects, FcγRIIB is also implicated in limiting responses to immune checkpoint blocking mAbs^30–32^ and, paradoxically, augmenting many immunostimulatory mAbs (eg, anti-CD40, -OX40, 4-1BB, and -DR5).^33–36^ This latter function is achieved through its ability to further cross-link mAb: antigen complexes and enhance target receptor clustering.^33^^,^^37–39^ These effects are isotype-dependent, with those exhibiting preferential FcγRIIB-binding (eg, mouse IgG1) the most potent agonists.^33^^,^^40^

In contrast to mouse FcγRII, human FcγRIIB appears more selectively expressed.^41^ FcγRIIB is broadly detectable in B-lymphocytes, monocytes, basophils, myeloid-derived dendritic cells and hepatic Kupffer cells,^4^^,^^41–43^ while other healthy tissue macrophages demonstrate a more variable pattern of expression.^41^^,^^44^ FcγRIIB expression is, however, frequently elevated in primary human tumours^23^^,^^45–47^ and metastatic sites^48^ including on tumour-associated monocyte and macrophage populations.^23^ Mirroring observations in the mouse, upregulation or dysregulation of human FcγRIIB is also associated with poor outcomes to tumour immunotherapy^45^^,^^49^ and development of IC-mediated autoimmune disease, in the form of systemic lupus erythematosus.^15^^,^^50–55^ Accordingly, human FcγRIIB elicits similar restraint of BCR- and FcγR-derived signals as well as *in vitro* mAb effector mechanisms.^5^^,^^13^^,^^29^^,^^56^^,^^57^ Human FcγRIIB is also capable of internalising certain cell surface mAb: antigen complexes (eg, CD20 and CD19)^58^ when engaged in *cis,* leading to removal of accessible mAb and reduction of Fc available to engage effector mechanisms.^19^^,^^58–61^ Consequently, FcγRIIB is also recognized as a critical negative regulator of mAb-mediated immunotherapy in humans.^45^^,^^49^

As biologically and therapeutically relevant FcγRIIB functions are frequently regulated through modulation of expression, a detailed understanding of the molecular regulators in multiple cell types is required. In the mouse, the polymorphic *fcgr2b* promoter^16^ contains putative transcription start site (TSS) proximal glucocorticoid response, E box, and S box elements alongside AP-1,^16^ AP-4,^55^ and SP1 transcription factor (TF) binding sites.^1^^,^^62^ However, a more robust role for the ETS-family member PU.1^63–67^ has been elucidated with additional and contrasting roles for the related factors SPIB^65^^,^^68^^,^^69^ and SPIC^66^ implied.

Despite similarities in promoter sequence between mouse and human,^64^ the identities of critical human TFs and promoter elements that drive FcγRIIB expression are poorly defined.^1^ In humans, Nishimura et. al identified a TSS proximal minimum required promoter fragment (−163: +59 bp from TSS) associated with ZNF140 and ZNF91-mediated repression under ectopic expression conditions.^70^ Additional studies have identified AP-1^54^ binding at position -304 and TSS proximal HIF2 binding under hypoxic conditions that co-operate to drive hypoxia-mediated FcγRIIB upregulation.^23^ As AP-1 binds outside the minimum required promoter fragment, the nature of critical TFs responsible for basal FcγRIIB promoter regulation remains unclear.

Given this ambiguity, we systematically dissected the human FcγRIIB promoter to identify critical regulatory elements and TF binding sites. Using human immune cells, we identify that TSS proximal PU.1 binding is essential for promoter activity in models of B-lymphocytes and monocytes. In B-cells, the related TF SPIB also exhibits redundancy with PU.1 in regulating FcγRIIB expression. As SPIB is not expressed in monocytes, these observations represent lineage-specific mechanisms of FcγRIIB promoter regulation. PU.1 is also essential for activating FcγRI and IIA expression in monocytes. However, in isolation, ectopic PU.1 expression is insufficient to drive FcγR expression. Consequently, PU.1 likely primes FcγR loci for transcription and requires co-operation with additional, potentially lineage-specific, TF to elicit FcγR expression.

## Materials and methods

### Human subjects and cell lines

Anonymized leukocyte cones were obtained from informed consenting healthy adult donors attending the Southampton Blood Donor Centre (National Health Service Blood & Transplant, Southampton, UK). The use of primary human material was reviewed and approved locally by the University of Southampton Faculty of Medicine Ethics Committee (19660.A11) and at a national level by the National Health Service/Health and Social Care Research Ethics Committee (REC) (IRAS: 186605). Peripheral blood mononuclear cells (PBMC) were isolated using Lymphoprep density gradient centrifugation medium (Stem Cell Technologies) as described previously.^23^ Primary human monocytes were isolated by magnetic separation using negative selection pan monocyte isolation kits or positive selection (Fig. 5A only) CD14 microbeads (both Miltenyi Biotech). Primary human B-cells were purified by positive selection using CD19 microbeads (Miltenyi Biotech). Purified monocytes were maintained in CTL test medium (CTL) supplemented with 1 mM pyruvate, 2 mM glutamine, 100 U ml^−1^ penicillin, and 100 µg ml^−1^ streptomycin at 1 × 10^6^ ml^−1^ in a humidified 37°C, 5% CO_2_ incubator_._ THP-1, Raji, and Ramos cell lines were obtained from the American Type Culture Collection (ATCC). BJAB and SUDHL6 were the generous gift of Prof. G. Packham. THP-1 cGAS^-/-^ cell line was obtained from Invivogen. All cell lines were maintained at 37°C, 5% CO_2_ in complete RPMI (RPMI 1640 (Thermo Fisher Scientific) supplemented with 1 mM pyruvate, 2 mM glutamine, 100 U ml^−1^ penicillin, 100 µg ml^−1^ streptomycin, 10% heat-inactivated foetal bovine serum (FBS)). HEK293F were obtained from Thermo Fisher Scientific and cultured in Freestyle293 medium (Thermo Fisher Scientific).

### Animals

Human (h)FcγRIIB^+/−^ C57BL/6 J mice, described previously,^5^ were maintained in local animal facilities in individually ventilated cages (IVC) under specific pathogen-free (SPF) conditions. The use of animals was approved by the University of Southampton Animal Welfare Ethical Review Board (AWERB) and was conducted under UK Home Office Project license P4D9C89EA. Experiments used both male and female mice at approximately 12 weeks of age. Groups were age- and sex-matched. Mice were maintained on a 12-hour light/dark cycle, with food and water available *ad libitum*. For in vivo administration, PLX51107 (Selleck Chemicals) was formulated in 10% n-methyl-2-pyrrolidone, 40% polyethylene glycol-400, 5% tocopherol methoxypolyethylene glycol succinate, and 5% Poloxamer 407, as previously described,^71^ and administered at 10 mg/kg *per os* by gavage once daily. Animals were monitored daily for adverse effects, with no toxicities apparent in the study. Animal tissues were extracted and disaggregated to form single cell suspensions as previously described.^23^

### Human FcγR TSS annotation and reporter assays

A representative TSS position per FcγR gene was determined from Ensembl v97 (July 2019) annotation. The representative TSS chosen has the greatest number of Ensembl Havana transcripts, else the most transcripts, else is the most 5′, and was implemented using CiiiDER.^72^ TSS coordinates are +1-based. The human FcγRIIB TSS was defined as 161,663,160 using the chromosome 1, GRCh38.p14 Primary Assembly (NC_000001.11), as outlined in Fig. S1A. Positional information, both from this study and the wider literature, have been updated to reflect this nomenclature. A human FcγRIIB 1 Kb promoter fragment (Table S1) was cloned from Raji genomic DNA, as described in,^5^ and ligated into pMCS Cypridina Luc (Thermo Fisher). Deletion constructs (Table S1) were created by using a site-directed mutagenesis approach. Polymerase chain reaction (PCR) primers were designed to flank the proposed deletion sites. PCR of the pMCS Cypridina Luc plasmid was performed using Phusion High-Fidelity DNA Polymerase (New England Biolabs). The PCR product was gel purified using QIAquick Gel Extraction Kit (Qiagen) and was subsequently treated with DpnI to destroy the original plasmid (New England Biolabs), phosphorylated using T4 Polynucleotide Kinase (New England Biolabs) and self-ligated overnight using T4 DNA Ligase (New England Biolabs). The reaction mixture was used for bacterial transformation using NEB^®^ 5-alpha Competent *E. coli* (New England Biolabs). Individual colonies were grown to isolate the plasmid, and deletions were confirmed by Sanger sequencing.

**Figure 1.**
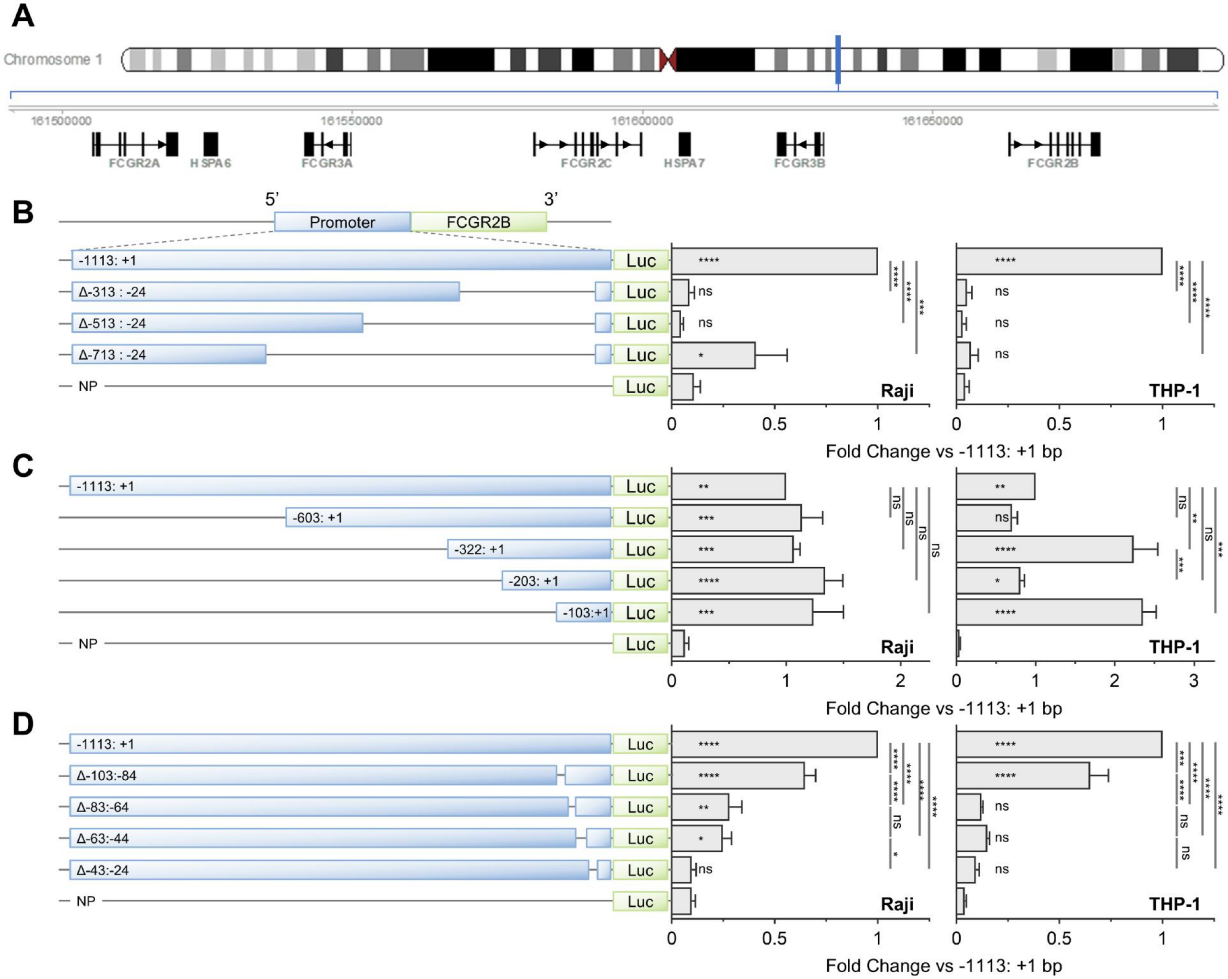
FcγRIIB promoter activity is dictated by TSS proximal sequence. (A) The structure of the low-affinity FcγR locus is depicted and its location within chromosome 1, in the region chr1:161,490,000–161,700,000, identified as a blue vertical line. (C, D) Raji or THP-1 cGAS^-/-^ cells were transfected with luciferase FcγRIIB promoter constructs with (B) 3′ deletions, (C) 5′ deletions, or (D) TSS proximal deletions and a constitutive internal control. Luciferase activity was assessed after 24 h, normalised to internal controls, and expressed as fold change from the unmodified −1113: +1 bp promoter construct. NP denotes no promoter control. Promoter diagrams depict to-scale demonstrations of deleted regions. Data denote the mean of at least 3 independent experiments each performed in triplicate. Error bars denote S.E.M. Statistical analyses were performed using 1-way ANOVA and adjusted for multiple comparisons via Tukey’s test. ns = non-statistically significant, *P < 0.05, **P < 0.005, ***P < 0.0005, ****P < 0.00005. In all figures, bars represent mean.

Raji, THP-1 cGAS^-/-^, Ramos, SUDHL6 and BJAB cell lines were transfected using the Neon Transfection System (Thermo Fisher Scientific). Prior to transfection, cells were grown in antibiotic-free RPMI1640 (Thermo Fisher Scientific) supplemented with 1 mM pyruvate, 2 mM glutamine, 10% FBS. Cells were seeded at 3 × 10^5^ ml^−1^ and 48 h later transfected using 100 µl (Raji, THP-1) or 10 µl tips (Ramos, BJAB, SUDHL6). Cells were washed with PBS and the supernatant removed following centrifugation (300×*g*, 5 min). Cell pellets were resuspended at 2 × 10^7^ ml^−1^ in Buffer R alongside 5 µg of the indicated pMCS Cypridina Luc construct and 5 µg pCMV Firefly Luc (Thermo Fisher Scientific) constitutive control construct. Cells were aspirated into Neon tips and electroporated using the following parameters: 1350 V, 30 ms, 1 pulse. Following electroporation, cells were seeded at 1 × 10^6^ ml^−1^ in antibiotic-free RPMI1640 (Thermo Fisher Scientific) supplemented with 1 mM pyruvate, 2 mM glutamine, 20% heat-inactivated FBS and grown in a humidified 37°C, 5% CO_2_ incubator. Luciferase activity was measured 24 h later using Pierce Cypridina-Firefly Luciferase Dual Assay Kit (Thermo Fisher Scientific) according to the manufacturer’s instruction and a Varioskan Flash microplate reader (Thermo Fisher Scientific). Background luminescence readings were subtracted from recordings and cypridina luciferase data normalized to the firefly luciferase internal control. Data were expressed as fold change from the full −1113: +1 bp pMCS Cypridina Luc construct. HEK293F cells were transfected with 10 µg pCMV3 or pCMV3 PU.1 (both Sino Biological) using Freestyle MAX transfection reagent (Thermo Fisher Scientific) according to the manufacturer’s instruction.

### Gene knockdown

Raji and THP-1 cell lines were transfected as described above except 200 picomoles gene-specific siRNA (Detailed in Table S2) or an appropriate scrambled siRNA control was utilised (Table S2). Impacts upon target gene expression were monitored 24 and 48 h later by qPCR and/or immunoblot. Impacts upon FcγRIIB expression were reported at timepoints exhibiting peak target gene knockdown (24 or 48 h). Isolated monocyte gene knockdown was similarly achieved using 100 µL tips, except cells were resuspended at 3 × 10^7^ ml^−1^ in buffer T and electroporated at 1920 V, 25 ms, 1 pulse. Post electroporation, monocytes were cultured at 1 × 10^6^ ml^−1^ in CTL medium supplemented with 1 mM pyruvate, 2 mM glutamine, 20% heat-inactivated FBS. Monocyte gene knockdown data are reported 48 h post transfection.

### Flow cytometry

Human cell lines or purified immune cells were incubated at room temperature with complete RPMI 1640 supplemented with 10% Human AB serum (Sigma Aldrich) for 15 min. Cells were centrifuged, the supernatant discarded, and resuspended in flow cytometry wash buffer (PBS, 1% BSA [Europa], 0.1% sodium azide [Sigma-Aldrich]) containing appropriate concentrations of fluorochrome-conjugated antibodies and stained for 30 min at 4°C. Samples were stained with anti-CD19 APC (clone: HIB19) or anti-CD14 Pacific Blue (clone: 63D3) (both Biolegend) according to the manufacturer’s recommendations in conjunction with FcγR-specific Fc-silenced mAbs. FcγR staining utilised in-house Alexa fluor 488-conjugated antibodies: anti-FcγRI (clone: 10.1, F(ab')2), anti-FcγRIIA (clone: E08 IgG1 N297Q), anti-FcγRIIB (clone: 6G11 IgG1 N297Q) in comparison to AT171-1 IgG1 N297Q or D10E6 mouse IgG1 F(ab)’_2_. FcγRIIA or IIB-specific mAbs were described previously^5^ and provided by BioInvent International AB, FcγRI-specific mAb was generated from published sequences. Samples were then washed with flow cytometry wash buffer, centrifuged at 340×*g*, supernatant discarded, and resuspended in 100 μl wash buffer. Samples were then acquired using flow cytometry on a FACSCanto II (BD Biosciences) and analyzed using FlowJo Software (BD Biosciences). Geometric mean fluorescence intensity (gMFI) was recorded and normalised to gMFI of an appropriate isotype control by subtraction (ΔgMFI).

### Chromatin immunoprecipitation

Chromatin was isolated from 4 × 10^6^ cells per preparation using the SimpleChIP^®^ Enzymatic Chromatin IP Kit (Cell Signalling Technology) according to the manufacturer’s instruction, as previously described.^23^ Chromatin was digested using 1000 units micrococcal nuclease per preparation at 37°C for 20 min and samples were sonicated using an S-3000 sonicator (Misonix) for 6 cycles of 15 s (power setting 1.5) followed by a 30 s rest. Nuclear lysis was confirmed by trypan blue staining and appropriate DNA fragmentation assessed by agarose gel electrophoresis. 10 µg chromatin was utilised per immunoprecipitation with optimised concentrations of target-specific or isotype control antibody (Table S3) incubated overnight at 4°C. Input control or immunoprecipitation samples were assessed for target enrichment by qPCR using site-specific primers (Table S4). qPCR was performed as previously described^73^ using either Taqman (Thermo Fisher Scientific) or Sybr-green chemistry using the 2^−ΔΔcT^ or absolute quantification for gene expression and ChIP analysis, respectively. Assay IDs and primer sequences are detailed in Tables S4–5. Data were acquired using a CFX connect instrument (Bio Rad) running CFX Maestro software (Bio Rad). Primer amplification efficiency calculation and data adjustment was performed utilizing standard curves of ChIP input material using on-board tools of the CFX Maestro Software (Bio Rad).

### Antibodies and reagents

Immunoblot analysis was performed using standard techniques, as previously described.^23^ ChIP antibodies outlined in Table S3 were utilized in addition to: anti-IRF3 (Cell Signalling Technology, #11904), anti-Actin (Cell Signalling Technology, #3700), and anti-FcγRIIB (Abcam, ab45143). DMOG was obtained from Sigma-Aldrich, BETi JQ-1 and PLX51107 were obtained from Selleck Chemicals. IL-6 and IL-10 were obtained from Peprotech.

## Results

Given the critical roles of FcγRIIB in modulating humoral immunity^10^^,^^17^ and therapeutic mAb activity,^20^ we aimed to identify promoter elements that underpin human FcγRIIB expression to address the lack of current understanding. We profiled the sequence 1 kb (−1113: +1 bp) upstream of the TSS (Fig. 1A–B & TSS defined in Fig. S1A) for regulatory function through a series of luciferase reporter constructs deleted for different regions of the 1 kb region transiently transfected into human B-lymphocyte and monocyte model systems (Table S1). Raji (B-lymphoma) and THP-1 (monocytic leukaemia) cell lines were selected due to previous reports describing putative differences in FcγRIIB promoter regulation.^70^ Promoter elements that retained the TSS and its flanking sequence but lacked the upstream regions −313: −24 or −513: −24 bp exhibited reporter activity indistinguishable from promoterless controls in both Raji and THP-1 (Fig. 1B). These data identify that sequence within 313 bp of the TSS exerts critical regulatory function. To further refine these observations, we employed 5′ promoter deletion constructs to define the minimum region required for promoter activity. A 104 bp (−103: +1 bp) TSS proximal sequence demonstrated maximal reporter activity indistinguishable from, or greater than, the remaining promoter fragments (Fig. 1C). When combined with 3′ deletion data (Fig. 1B), these observations define the critical regulatory elements as −103: −24 bp from TSS. To further improve resolution, we subsequently modified the full length −1113: +1 bp reporter construct to incorporate ≈20 bp deletions within the −113: −24 bp TSS proximal sequence. Reporter activity was significantly reduced by disruption of any sequence located within this region; most profoundly following deletions within −83: −24 bp of TSS (Fig. 1D). These collective observations were also apparent in 3 additional human B-cell lymphoma model systems (Fig. S1B–D) confirming that modification of a 60 bp region located between −83: −24 bp from TSS is highly detrimental to promoter activity, indicative of critical regulatory function.

**Figure 2.**
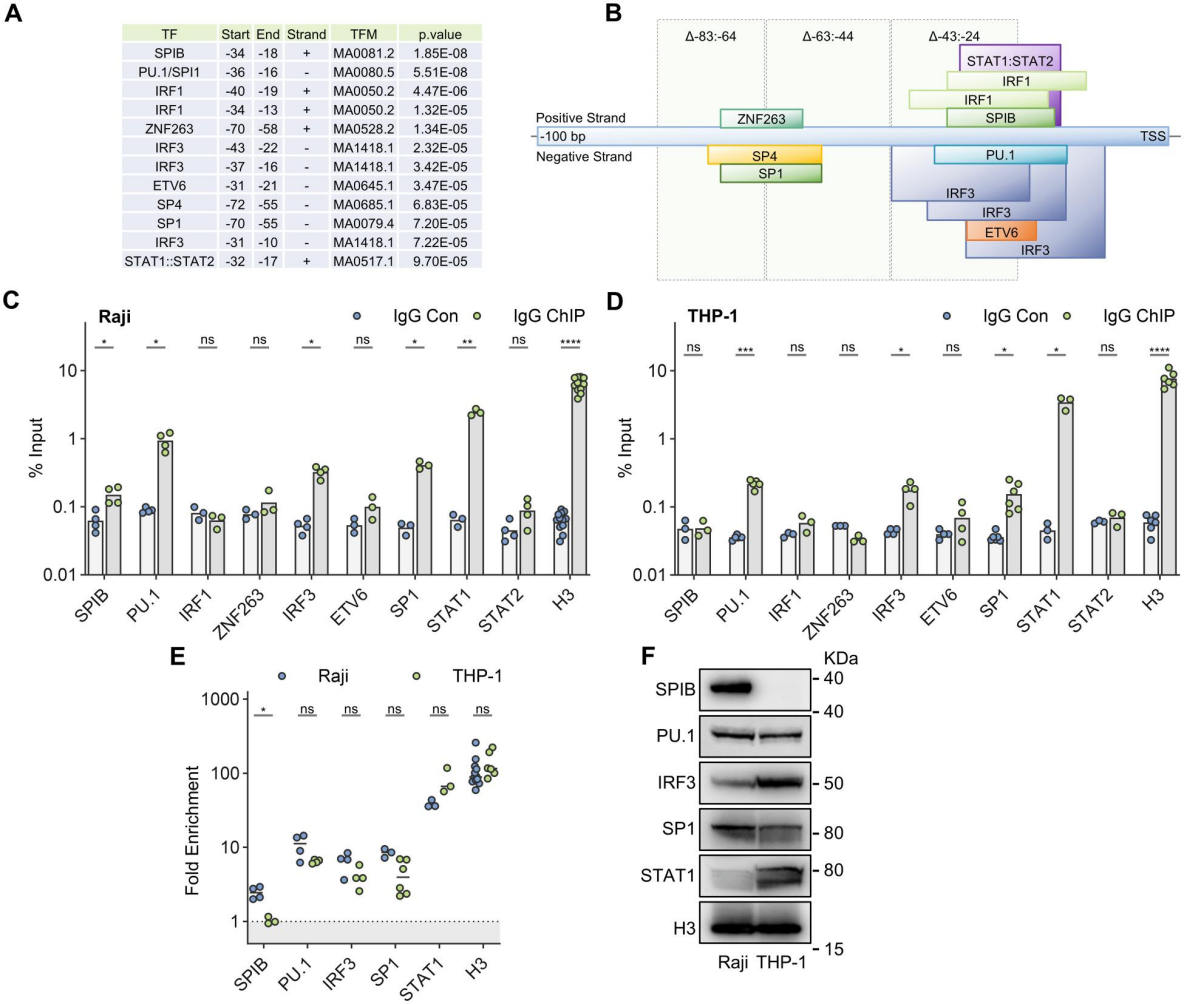
FcγRIIB TSS proximal sequence is associated with candidate TF binding. (A, B) Sequence 1.5 kb upstream and 0.5 kb downstream of the FcγRIIB TSS was profiled for putative TF recognition motifs using the FIMO algorithm and position frequency matrices of the human JASPAR 2020 CORE collection. Statistically significant motifs (*P* < 0.0001) were filtered for positive expression in human B-cell, monocyte, or macrophage RNA seq data sets and location within the 0.1 kb TSS upstream region. Filtered TF motifs, their positional data, and *P* values are tabulated in (A) and spatially represented in (B) with deleted regions from Fig. 1C) highlighted. (C, D) FcγRIIB TSS proximal sequence was profiled for candidate TF binding by ChIP using TF-specific mAbs (IgG ChIP) or an appropriate IgG control (IgG Con) in (C) Raji and (D) THP-1 cells. Data points represent independent experiments, each performed in triplicate. TF enrichment is expressed as % input and compared to a Histone H3 immunoprecipitation control (H3). (E) ChIP data from (C, D) expressed as fold enrichment over IgG control and compared across cell lines. (F) Immunoblot analysis of whole cell protein isolates from Raji and THP-1 cell lines. Statistical analysis was performed via multiple paired (C, D), or unpaired (E) T-tests corrected for multiple comparisons using the Holm-Sidak method. ns = non-statistically significant, *P < 0.05, **P < 0.005, ****P < 0.00005. In all figures, bars represent mean.

To identify putative TF binding sites within this TSS proximal region, we employed the predictive motif-matching FIMO algorithm (MEME suite version 5.4.1),^74^ using the position frequency matrices of the human TF JASPAR 2020 CORE collection^75^ of TF motifs to profile the 2 kb FcγRIIB promoter sequence (1.5 kb upstream of TSS and 0.5 kb downstream). Expression of genes encoding statistically significant motifs (*P* < 0.0001) was assessed and filtered for positive (≥5 TPM) expression in human B-lymphocytes, monocytes, or macrophages using publicly available datasets (Human protein atlas (www.proteinatlas.org) or Blueprint).^76^^,^^77^ We identified 12 predicted TF motifs within −83: +1 from the TSS forming two distinct clusters (Fig. 2A, B). Critically, the most TSS proximal TF cluster was bisected by the Δ−43: −24 deletion responsible for the most profound suppression of reporter activity (Fig. 1D and 2B). To experimentally identify TSS proximal binding of these candidate TF, we performed FcγRIIB TSS-targeted chromatin immunoprecipitation (ChIP) qPCR analysis in Raji and THP-1 cells. Notably, similar patterns of TF enrichment were observed at FcγRIIB TSS proximal locations, despite differences in cell lineage, with PU.1, IRF3, SP1, and STAT1 exhibiting binding in both cell lines (Fig. 2C–E). In contrast, association of SPIB with FcγRIIB TSS proximal sequence was specific to Raji (Fig. 2C–E). SP4 binding could not be assessed due to a lack of available ChIP-grade antibodies at the time of study. FcγRIIB TSS-associated TF were expressed in both cell lines to variable degrees with the exception of SPIB, which was undetectable in THP-1 cells (Fig. 2F). Consequently, differences in SPIB TSS binding correlated with differences in expression level between the cell lines. In contrast, the remaining factors demonstrated a similar extent of TSS proximal binding between cell lines despite variable expression levels (Fig. 2E, F).

**Figure 3.**
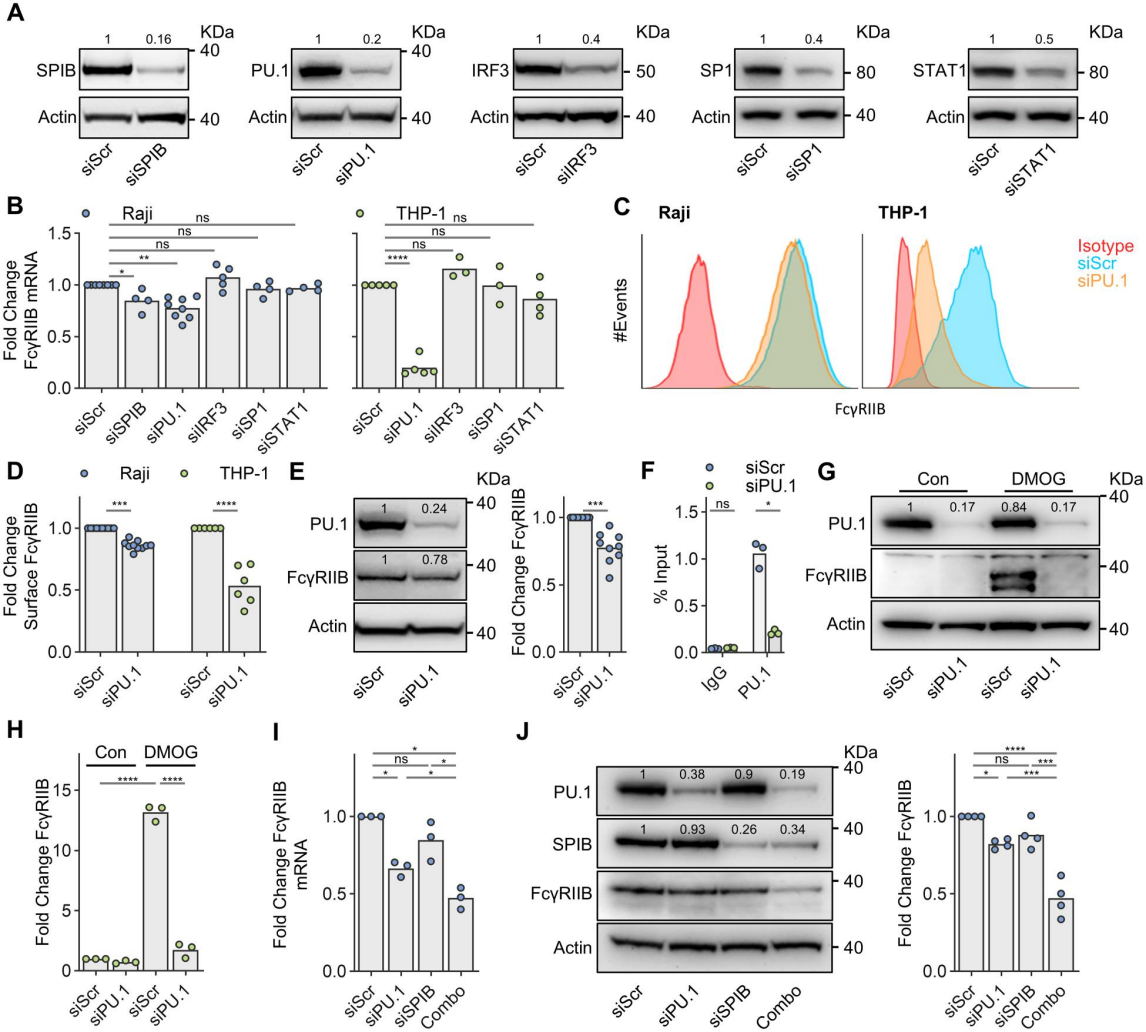
PU.1 and SPIB redundantly co-regulate FcγRIIB expression in a cell-specific manner. (A) The impact of FcγRIIB TSS-associated TF gene knockdown upon target protein expression was assessed by immunoblot in Raji cells. Blots are representative examples of at least 3 independent experiments. (B) The impact of FcγRIIB TSS-associated TF gene knockdown upon FcγRIIB transcript levels was assessed by qPCR in Raji (blue) or THP-1 (green) cells following transfection with gene-specific siRNA in comparison to a scrambled siRNA control (denoted as siSPIB, siPU.1, siIRF3, siSP1, siSTAT1, and siScr, respectively). Data are expressed as fold change from siScr, points represent independent experiments each performed in triplicate. (C, D) The impact of PU.1 knockdown upon FcγRIIB surface protein levels of Raji and THP-1 cells was assessed by flow cytometry, with representative flow cytometry data depicted in (C). Data points in (D) represent independent experiments. (E) The impact of PU.1 knockdown was assessed by immunoblot analysis in Raji cells. A representative immunoblot is depicted with densitometry data of 8 independent experiments adjacent. (F) THP-1 cells transfected with siPU.1 or siScr were assessed by PU.1-directed ChIP in comparison to an isotype control (IgG) using FcγRIIB TSS-specific primers and expressed as % input. A representative example of 2 independent experiments is demonstrated. Data points represent triplicates. (G, H) The impact of PU.1 knockdown upon inducible FcγRIIB expression was assessed in THP-1 cells by immunoblot. PU.1 targeted siRNA (siPU.1) or a scrambled siRNA control (siScr) were delivered 24 h prior to addition of 1 µM DMOG or a DMSO vehicle control (Con) for a further 24 h. A representative immunoblot is depicted in (G) with densitometry data from 3 independent experiments in (H). (I, J) The impact of PU.1 and SPIB gene co-silencing upon FcγRIIB expression was assessed in Raji cells following transfection with PU.1- (siPU.1) or SPIB-targeting (siSPIB) siRNA alone or in combination (Combo) compared to a scrambled siRNA control (siScr). FcγRIIB expression was assessed at the transcript level (I) by qPCR and at the protein level (J) by immunoblot. qPCR data represents the mean of 3 independent experiments. A representative immunoblot (left) is depicted in (J) alongside densitometry data (right) from 3 independent experiments. Statistical analysis was performed using mixed model (B), 1-way (F, H), or 2-way (D, F) ANOVA adjusted for multiple comparisons using Dunnett’s, Sidak’s, or Tukey’s test. (E) was assessed using Wilcoxon matched-pairs signed rank test. ns = non-statistically significant, *P < 0.05, **P < 0.005, ***P < 0.0005, ****P < 0.00005. In all figures, bars represent mean.

Subsequently, we employed RNAi-mediated gene knockdown to determine the roles of these candidate TF in driving FcγRIIB expression. Gene knockdown was assessed in both Raji and THP-1 cells at the protein and transcript levels (Fig. 3A and Fig. S2A, B). PU.1 knockdown elicited a modest reduction in baseline FcγRIIB transcript, surface protein, and total protein levels in Raji cells (Fig. 3B–E). In contrast, more robust reductions in FcγRIIB expression were observed following PU.1 knockdown in THP-1 cells (Fig. 3B–D). ChIP analysis determined that PU.1 appeared enriched at FcγRIIB TSS proximal locations in comparison to distal/intronic regions (Fig. S3A, B) and that PU.1 knockdown reduced the level of PU.1 associated with FcγRIIB TSS proximal sequence (Fig. 3F). Subsequently, we investigated the impact of PU.1 knockdown upon hypoxia induced FcγRIIB upregulation in THP-1 cells, as previously described.^23^ PU.1 expression appeared largely unaltered by the HIF prolyl hydroxylase inhibitor, and hypoxia mimetic, DMOG and following incubation under hypoxic conditions (Fig. 3G & Fig. S3C). However, PU.1 gene knockdown effectively ablated FcγRIIB upregulation at both the transcript and protein levels (Fig. 3G, H & Fig. S3C–E). Thus, PU.1 appears to play a prominent role in baseline and inducible FcγRIIB expression in the monocytic THP-1 cell line, with more modest effects upon baseline expression observed in the Raji B-cell line. Similar to the limited impact of PU.1-targeting siRNA, knockdown of the related ETS-family member SPIB modestly reduced FcγRIIB levels in Raji cells (Fig. 3B). Given that THP-1 cells lack SPIB expression and demonstrate an increased impact of PU.1 knockdown upon FcγRIIB expression, we reasoned that SPIB and PU.1 may exhibit functional redundancy in Raji cells and, therefore, mask effects upon FcγRIIB expression. Accordingly, co-silencing of PU.1 and SPIB expression elicited a more robust reduction in FcγRIIB at both the transcript and protein levels in Raji cells (Fig. 3I, J). In contrast, silencing of other FcγRIIB TSS associated TF (IRF3, SP1, and STAT1) failed to elicit any detectable impact upon FcγRIIB expression following gene knockdown (Fig. 3B) or any additional effect when combined with PU.1 gene silencing (data not shown). Consequently, the ETS-family member TF PU.1 appears essential for FcγRIIB expression in the monocytic cell line THP-1, whereas, in Raji cells, PU.1 appears to co-operate in a redundant fashion with SPIB.

**Figure 4.**
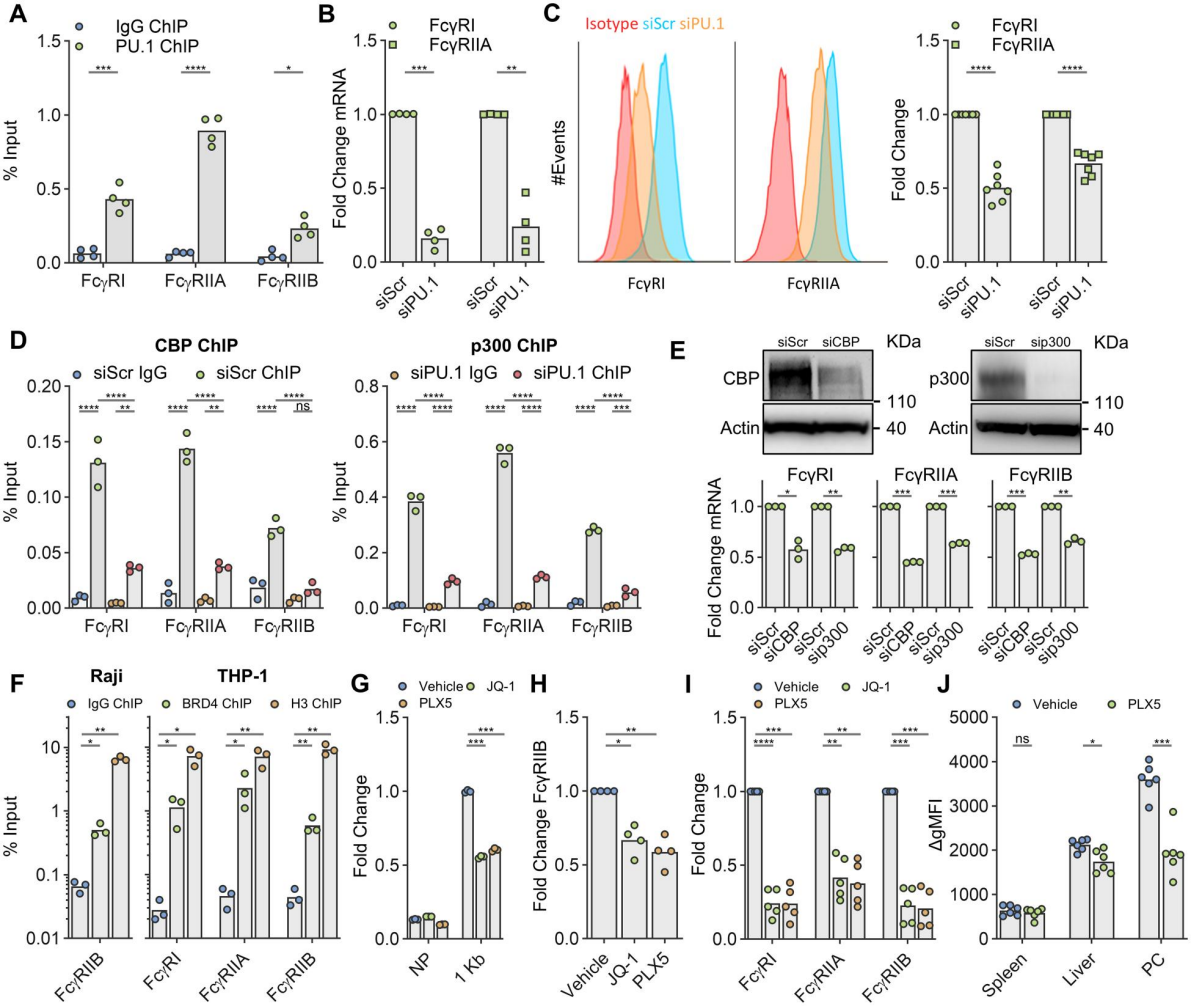
PU.1 regulates FcγR expression through CBP/p300 and BRD4. (A) PU.1 association with FcγRI, FcγRIIA, or FcγRIIB TSS proximal sequence was assessed in THP-1 cells by ChIP (PU.1 ChIP) versus an isotype control (IgG ChIP). Data points represent independent experiments, each performed in triplicate, normalised for primer amplification efficiency, and expressed as % input. (B, C) The impact of PU.1 knockdown upon FcγRI and FcγRIIA expression was observed in THP-1 cells at the (B) transcript and (C) surface protein levels by qPCR and flow cytometry, respectively. (C) (left and center) depicts representative flow cytometry data; right, fold change from multiple donors. Data points represent independent experiments. (D) CBP or p300 association with FcγRI, FcγRIIA, or FcγRIIB TSS proximal sequence was assessed by ChIP in THP-1 cells as in (A) 48 h post transfection with PU.1-targetted siRNA (siPU.1) or a scrambled siRNA control (siScr). Points represent triplicate values. Data is representative of 2 independent experiments. (E) FcγR transcript levels were assessed in THP-1 cells by qPCR performed in triplicate following CBP/p300 (siCBP, sip300) gene silencing in comparison to a scrambled siRNA control (siScr); blots above represent extent of target knock-down. (F) BRD4 association with FcγR TSS proximal sequence assessed by ChIP in Raji (left) or THP-1 (right) cells in comparison to isotype (IgG ChIP) and Histone H3 (H3 ChIP) controls. Points represent independent experiments each performed in triplicate. (G) Raji transiently transfected with −1113: +1 bp FcγRIIB promoter, or no promoter control (NP), luciferase reporter constructs were subjected to 250 nM JQ-1, 625 nM PLX51107 (PLX5), or a vehicle control for 24 h. Reporter activity was assessed and normalised as in Fig. 1A. Points represent triplicates. (H, I) Raji (H) or THP-1 (I) cells were treated with JQ-1 or PLX51107 (PLX5) as outlined in (G) for 24 h and FcγR surface protein levels assessed by flow cytometry, presented as fold change from vehicle-treated cells. Data points represent independent experiments. (J) hFcγRIIB Tg^+/−^ mice were treated daily with 10 mg/kg PLX51107 (PLX5) or a vehicle control *per os* for 5 d. Mice were sacrificed 4 h post final dose and peritoneal cavity (PC), spleen, and liver monocytes (CD11b^+^, Ly6C^+^, Ly6G^−^, F4/80^−^) were assessed for hFcγRIIB expression by flow cytometry. Geometric means (MFI) were normalised (via subtraction) to an appropriate isotype control (ΔgMFI). Data points represent individual animals. In all figures, bars represent mean. Statistical analyses were performed via *T*-tests (A, J) adjusted for multiple comparisons using the Holm-Sidak method or 1- (E, F, H, I) or 2-way (B–D, G) ANOVA adjusted for multiple comparisons using Sidak’s (B, C, E), Tukey’s (D), or Dunnett’s (F, G) tests. ns = non-statistically significant, *P < 0.05, **P < 0.005, ***P < 0.0005, ****P < 0.00005.

Given the essential roles of PU.1 in haematopoietic lineage commitment and regulation of a multitude of immune function-associated target genes, we additionally profiled TSS proximal regions of activating FcγR loci (FcγRI, FcγRIIA, FcγRIIIA, FcγRIIIB) for TF binding motifs. Similar to FcγRIIB, FcγRI and FcγRIIA promoters exhibit putative PU.1 binding motifs located within 50 bp of TSS (Fig. S4A–D) and demonstrate TSS proximal PU.1 binding, as assessed by ChIP (Fig. 4A). In contrast, FcγRIIIA/B promoters lack TSS proximal PU.1 binding motifs (data not shown). Accordingly, PU.1 knockdown elicited a profound reduction in both FcγRI and FcγRIIA transcript and surface protein levels in THP-1 cells (Fig. 4B, C). Similar to FcγRIIB these effects were also concordant with depletion of PU.1 from TSS proximal sequences following PU.1 knockdown (Fig. S5A). Consequently, PU.1 appears to not only contribute toward inhibitory FcγRIIB expression but also that of the activating FcγRI and FcγRIIA in THP-1 cells. As THP-1 cells lack detectable FcγRIIIA/B expression, the impact of PU.1 knockdown upon these FcγR (that lack TSS proximal PU.1 motifs) remains to be determined.

**Figure 5.**
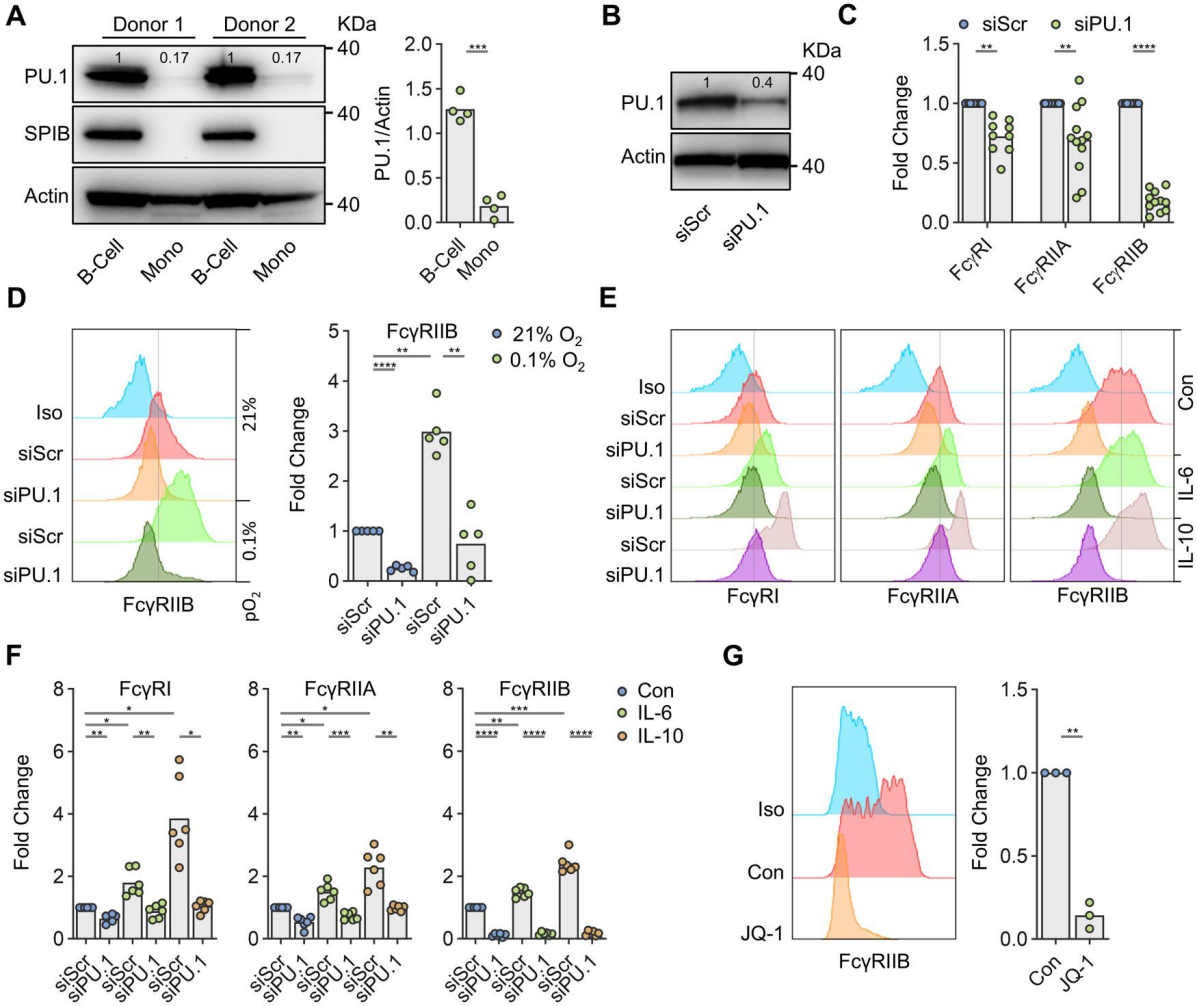
PU.1 regulates FcγR expression in primary human monocytes. (A) B-cells and monocytes (Mono) purified from PBMC were profiled for PU.1 and SPIB expression by immunoblot. A representative immunoblot is demonstrated alongside densitometry data obtained from 4 donors. (B, C) Purified monocytes transfected with PU.1 targeting siRNA (siPU.1) or a scrambled siRNA control (siScr) were assessed for expression of (B) PU.1 by immunoblot or (C) surface FcγR by flow cytometry in comparison to an appropriate isotype control. Data in (C) were normalised to siScr treated cells and expressed as fold change. (D) Purified monocytes subjected to siPU.1 or siScr for 24 h were cultured at either 21% or 0.1% O_2_ for a further 24 h. FcγRIIB levels were then assessed by flow cytometry and presented as fold change from siScr treated cells cultured at 21% O_2._ (D) left) depicts representative flow cytometry plots with data from individual donors summarised in (D) right). (E, F) PU.1 knockdown of purified monocytes was instigated, as in (D), and then treated with 50 ng/ml IL-6, 50 ng/ml IL-10, or a PBS vehicle control (Con) for a further 24 h. Surface FcγR expression was assessed by flow cytometry as in (D). (E) depicts representative flow cytometry plots, with data from individual donors summarised in (F). (G) Monocytes were cultured in the presence of 250 nM JQ-1 for 24 h and FcγRIIB expression level assessed by flow cytometry. (G) left) depicts representative histograms with data from individual donors summarised in (G) right). In all experiments bars represent mean. Statistical analyses were performed via paired *T*-tests (A, C, G) adjusted for multiple comparisons using the Holm-Sidak method where appropriate or one-way ANOVA (D, F) adjusted for multiple comparisons using Sidak’s test. ns = non-statistically significant, *P < 0.05, **P < 0.005, ***P < 0.0005, ****P < 0.00005.

Despite commonalities between FcγRI, FcγRIIA, and FcγRIIB regulation, additional mechanisms must also determine inhibitory/activating FcγR expression patterns, as B-lymphocytes lack activating FcγR expression. In order to determine whether PU.1 in isolation was sufficient to drive FcγR expression, we introduced exogenous PU.1 into HEK293F cells that under basal conditions express neither PU.1 nor FcγR. Ectopic expression resulted in enrichment of PU.1 at FcγRI, FcγRIIA, and FcγRIIB TSS proximal sequences and small yet significant increases in FcγR transcripts (Fig. S5B–D). However, despite supraphysiological PU.1 expression, FcγR protein could not be detected. Consequently, additional potentially cell lineage-specific factors are also required for FcγR expression.

In order to gain insight into the mechanisms of PU.1-mediated FcγR regulation, we investigated the impact of PU.1 gene knockdown upon recruitment of PU.1 interaction partners to FcγR TSS proximal locations. The prominent transcriptional co-activators and histone acetyltransferases (HAT), and known PU.1 interaction partners,^78^^,^^79^ CBP/p300 were enriched at FcγRI, FcγRIIA, and FcγRIIB TSS proximal sequences and were diminished following PU.1 knockdown in THP-1 cells (Fig. 4D). Importantly, PU.1 knockdown did not impact total protein levels of CBP or p300 (Fig. S5E). Knockdown of either CBP or p300 similarly reduced FcγRI, FcγRIIA, and FcγRIIB transcripts but did not influence PU.1 expression (Fig. 4E and Fig. S5F). Consequently, PU.1 appears to drive FcγR expression, at least in part, by recruitment of CBP/p300 to TSS proximal sequences. In addition to CBP/p300, we also assessed the contribution to FcγR expression of the potential PU.1 interaction partner,^67^ epigenetic reader, and bromodomain and extra-terminal (BET) family member BRD4. Similar to CBP/p300, BRD4 was enriched at FcγR TSS proximal sequences (Fig. 4F) in both Raji and THP-1 cells. To explore the impact of BRD4 recruitment on FcγR expression, we employed the bromodomain-specific BET family inhibitors (BETi) JQ-1 and PLX51107.^71^^,^^80^^,^^81^ BETi roughly halved FcγRIIB reporter activity in Raji cells (Fig. 4G) and reduced surface FcγR expression (Fig. 4H, I) in both Raji and THP-1 cells. Notably, the impact of BETi appeared more substantial in THP-1 cells and similarly affected activating and inhibitory FcγR. Given these observations, we examined the impact of BETi upon in vivo monocyte FcγRIIB expression through use of human FcγRIIB Tg mice, which express human FcγRIIB under the control of the human -1113: +1 bp TSS proximal sequence.^5^ BETi administration resulted in reduced hFcγRIIB levels in tissue monocytes of the liver and peritoneal cavity but not that of the spleen (Fig. 4J). Collectively, these data suggest known PU.1 interaction partners CBP/p300 and BRD4 contribute toward regulation of both activating and inhibitory FcγR.

To translate our findings into primary human immune cells, we contrasted PU.1/SPIB expression patterns of purified B-cells and monocytes. Consistent with cell line data, primary human B cells expressed PU.1 and SPIB. In contrast, monocytes demonstrated a lack of detectable SPIB and lower, yet consistently detectable, PU.1 expression than B-cell comparators (Fig. 5A). PU.1 gene knockdown significantly reduced basal surface FcγRI, FcγRIIA, and FcγRIIB expression in primary human monocytes (Fig. 5B, C). In contrast to THP-1 cells, approximately 5% to 10% of monocytes (non-classical) also express FcγRIIIA. Consistent with a lack of TSS proximal PU.1 motifs at this locus, PU.1 knockdown did not influence the frequency of FcγRIIIA^+^ monocytes (Fig. S6A).

Similar to our cell-line studies, human monocyte PU.1 expression levels appeared unaffected by hypoxia or the FcγR regulating cytokines IL-6^82^ or IL-10^83^^,^^84^ (Fig. S6B,C). However, PU.1 gene knockdown effectively ablated hypoxia-induced upregulation of FcγRIIB (Fig. 5D) and cytokine-mediated upregulation of FcγRI, FcγRIIA, and FcγRIIB in primary human monocytes (Fig. 5E, F and Fig. S6B,C). Further correlating with our cell-line data, BETi also effectively reduced basal FcγRIIB expression levels in primary human monocytes (Fig. 5G).

Collectively, our data identify PU.1, SPIB, IRF3, SP1, and STAT1 as FcγRIIB TSS proximal binding TFs in human immune cells. Of these, only PU.1 appears essential for basal FcγRIIB gene expression in both monocytes and B-cells. However, PU.1 also redundantly cooperates with the additional ETS family member SPIB in the Raji B-cell-line. Moreover, we identify conserved TSS proximal PU.1 binding as a fundamental cross cell-type determinant of both activating (FcγRI and FcγRIIA) and inhibitory (FcγRIIB) FcγR expression in humans. Mechanistically, PU.1 appears to recruit additional members of the core transcriptional machinery and epigenetic modifiers/readers, namely CBP/p300 and BRD4. However, PU.1 is insufficient to drive high level FcγR expression in an inappropriate cellular context. Furthermore, PU.1 expression appears largely insensitive to tissue/microenvironment-derived FcγR regulatory stimuli. Consequently, PU.1 appears to set the potential for FcγR expression with co-operation from additional lineage-specific and/or inducible factors required to drive FcγR expression.

## Discussion

Despite its critical roles in regulating humoral immunity,^9^^,^^10^^,^^13^ FcγRIIB poses a significant obstacle for mAb-mediated immunotherapy of cancer, due to frequent upregulation in both mouse^20–22^^,^^30^^,^^48^ and human^45^^,^^46^^,^^49^^,^^85^ tumors. Accordingly, FcγRIIB blocking mAbs have been developed that potentiate responses to immunotherapy in experimental mouse models.^5^ Building upon these findings, the impact of clinical biosimilars upon responses to established mAb immunotherapies are currently under evaluation (NCT03571568, NCT04219254), showing promising interim findings.^86–89^

As critical FcγRIIB functions often rely upon gene upregulation,^13^^,^^16^ a fundamental understanding of the regulatory mechanisms that dictate its expression are paramount. In the mouse, the ETS family TFs PU.1, SPIB, and SPIC have been implicated in FcγRIIB promoter regulation.^64–66^ Direct binding of PU.1 to the mouse FcγRIIB promoter has been observed experimentally,^64^ while significant decreases in FcγRIIB expression were also evident in mice with reduced PU.1 expression.^64^^,^^65^

In mice, PU.1 is broadly expressed within progenitor and mature hematopoietic cells, with its expression level a key determinant of the hematopoietic lineage adopted.^90–92^ Germline and conditional knockout studies have identified PU.1 as a master regulator of haematopoiesis^93^^,^^94^ responsible for regulation of myriad immune-associated target genes.^65^^,^^95^^,^^96^ Indeed, PU.1 is implicated in regulation of nearly all myeloid gene promoters lacking a TATA box through recruitment of TATA binding protein (TBP), CBP/p300, BRD4 and other components of the basal transcriptional machinery.^67^^,^^78^^,^^79^ PU.1 is capable of pioneer TF activity^97^^,^^98^ but may also act alongside additional factors at low affinity sites that require a pre-prepared chromatin context for transactivation.^95^^,^^98^

In murine B cells, SPIB appears to co-operate with PU.1 to transactivate and drive FcγRIIB expression.^65^ As a consequence of this functional redundancy, the latter stages of murine B-lymphopoiesis are largely unaffected by PU.1 deletion.^99^^,^^100^ Instead, combined loss of PU.1 and SPIB is required to reveal functional defects in mature B-cells.^65^^,^^101^ Despite this, SPIB knock-in into the PU.1 locus is unable to rescue the arrest in murine B-lymphopoiesis associated with loss of PU.1 function.^102^ Consequently, although SPIB and PU.1 redundancy is evident in driving mature B-cell function, they are clearly non-redundant during early lymphopoiesis.^102^

Prior to the current study, the relevance of PU.1 and SPIB to human FcγRIIB promoter regulation were unclear. Nevertheless, the high similarity of PU.1 recognition motifs and FcγRIIB TSS proximal sequence between mouse and humans were highly suggestive.^64^ Previously, a minimal promoter fragment (−163: +59 from TSS) required for human FcγRIIB promoter activity was identified; however, no positive regulatory factors associated with this region were determined.^70^ Olferiev et al. identified an AP-1 binding site at position −304, as a critical positive regulator of FcγRIIB.^54^ However, given its location outside the minimal promoter region, AP-1 binding alone is unlikely sufficient for FcγRIIB transactivation.^70^ Instead, it likely requires additional TSS proximal factors to facilitate transcription.

In the present study, we refined the human minimal required promoter region to positions -103: +1 in multiple immune models and demonstrated SPIB, PU.1, IRF3, SP1, and STAT1 TF binding in this region. Nishimura et al.^70^ previously discounted PU.1 binding to this region in Raji and THP-1 cells based on EMSA supershift assays. However, the conserved PU.1 recognition motif is located approximately 5 bp outside the boundaries of the regions probed in that study.^64^^,^^70^ Using PU.1-targeting siRNA, we observed a significant reduction in FcγRIIB expression in human cell lines and primary monocytes. This decrease was associated with loss of PU.1 from FcγRIIB TSS proximal sequence and a concurrent reduction in the recruitment of CBP/p300 transcriptional co-activators. Importantly, siRNA-mediated knockdown of CBP/p300 also resulted in reduced FcγRIIB expression, independently of PU.1 expression. This suggests that PU.1 promotes FcγRIIB transactivation, at least in part, by CBP/p300 recruitment. Furthermore, we identified BRD4 enrichment at the FcγRIIB TSS and its inhibition via BET inhibitors JQ-1^80^ and PLX51107^71^^,^^81^ led to a reduction in FcγRIIB promoter activity and expression both *in vitro* and *in vivo*. Although PU.1 knockdown substantially reduced FcγRIIB expression in THP-1 cells and monocytes, the effect was comparatively modest in the Raji B-cell model. This was attributable to functional redundancy with the related ETS-family member SPIB, highly expressed in human B-cells and plasmacytoid dendritic cells but not monocytes.^103^

AP-1 has also been implicated in driving both basal^54^ and hypoxia-inducible^23^ human FcγRIIB expression through promoter binding, despite location outside the minimally required FcγRIIB promoter region.^70^ As PU.1-targetted siRNA ablated both basal and hypoxia-inducible FcγRIIB expression in monocytes, it is possible that AP-1 cooperates with PU.1 to aid FcγRIIB transactivation. Interestingly, a co-operative relationship, and physical interaction, has been established between PU.1 and c-JUN (an AP-1 constituent) that is required for myeloid development in mice.^104^ The relevance of these events to human myelopoiesis remain to be determined. In addition to FcγRIIB, PU.1 was also enriched at TSS proximal sites, and required for gene expression, of the activating FcγRI and FcγRIIA as previously described/predicted.^105–107^ Mechanistically, this also appeared dependent upon recruitment of CBP/p300 to TSS proximal locations.

While the role of PU.1 in haematopoiesis is well defined in the mouse, evidence for an equivalent role in humans is less clear. However, a PU.1 haploinsufficiency-associated agammaglobulinemia was recently described where affected patients lack circulating B-lymphocytes and exhibit deficiencies in myelomonocytic populations.^108^ Consequently, PU.1 may also demonstrate analogous functions in human haematopoiesis.

While these collective observations represent significant progress in our understanding of human FcγR regulation, our study is limited by a lack of assessment of the functional implications of these critical regulatory elements upon immune physiology. Furthermore, as activating FcγR are not expressed by B-cells, how cell lineage-specific activating/inhibitory FcγR expression is regulated remains unclear. Although PU.1 is critical for FcγR expression in lymphoid/myeloid models, it is insufficient in isolation to drive expression in ectopic expression models such as HEK cells. Consequently, additional, potentially lineage-specific, factors or chromatin remodelling likely contribute to human FcγR expression regulation through cooperation with PU.1. This requirement may also underpin the variability seen in human FcγRIIB expression within tissue macrophage subsets.^41^

Here we identify that TSS-proximal PU.1 promoter binding is an essential determinant of basal and inducible human FcγR expression. It is anticipated that this basic understanding will form a foundation upon which future developments can build to understand the complexities of cell type-specific FcγR expression patterns and regulation. As our comprehension of the FcγR requirements that govern effective mAb therapy evolve and begin to influence their design,^40^ a complementary understanding of the molecular features that govern FcγR expression within tumours is paramount. This increased understanding may be leveraged to optimally deliver mAb therapeutics of the required isotype to elicit desired biological effects or to allow development of complementary drug combination strategies to potentiate mAb-mediated immunotherapy.

## Supplementary Material

vkaf109_Supplementary_Data

## Data Availability

The data sets used and/or analyzed during the current study are available from the corresponding author on reasonable request.

## References

[1] Smith KGC , Clatworthy MR. FcγRIIB in autoimmunity and infection: evolutionary and therapeutic implications (vol 10, pg 328, 2010). Nat Rev Immunol. 2010;10:674. 10.1038/nri2762 PMC4148599 20414206

[2] Roghanian A , Cragg MS, Frendéus B. Resistance is futile: targeting the inhibitory FcgγRIIB (CD32B) to maximize immunotherapy. Oncoimmunology. 2016;5:e1069939. 27057434 10.1080/2162402X.2015.1069939 PMC4801439

[3] Roghanian A , Stopforth RJ, Dahal LN, Cragg MS. New revelations from an old receptor: immunoregulatory functions of the inhibitory Fc gamma receptor, FcRIIB (CD32B). J Leukocyte Biol. 2018;103:1077–1088. 10.1002/JLB.2MIR0917-354R 29406570

[4] Kerntke C , Nimmerjahn F, Biburger M. There is (Scientific) strength in numbers: a comprehensive quantitation of Fc gamma receptor numbers on human and murine peripheral blood leukocytes. Front Immunol. 2020;11:118. 32117269 10.3389/fimmu.2020.00118 PMC7013094

[5] Roghanian A et al Antagonistic human FcγRIIB (CD32B) antibodies have anti-tumor activity and overcome resistance to antibody therapy in vivo. Cancer Cell. 2015;27:473–488. 25873171 10.1016/j.ccell.2015.03.005

[6] Ravetch JV , Kinet JP. Fc-receptors. Annu Rev Immunol. 1991;9:457–492. 1910686 10.1146/annurev.iy.09.040191.002325

[7] van der Poel CE et al Follicular dendritic cells modulate germinal center B cell diversity through FcγRIIB. Cell Rep. 2019;29:2745–2755.e4. 31775042 10.1016/j.celrep.2019.10.086 PMC7015177

[8] Ganesan LP et al FcγRIIb on liver sinusoidal endothelium clears small immune complexes. J Immunol. 2012;189:4981–4988. 23053513 10.4049/jimmunol.1202017 PMC4381350

[9] Takai T , Ono M, Hikida M, Ohmori H, Ravetch JV. Augmented humoral and anaphylactic responses in Fc gamma RII-deficient mice. Nature. 1996;379:346–349. 8552190 10.1038/379346a0

[10] Bolland S , Ravetch JV. Spontaneous autoimmune disease in FcγRIIB-deficient mice results from strain-specific epistasis. Immunity. 2000;13:277–285. 10981970 10.1016/s1074-7613(00)00027-3

[11] Muta T et al A 13-Amino-Acid motif in the cytoplasmic domain of Fc-Gamma-Riib modulates B-cell receptor signaling (Vol 368, Pg 70, 1994). Nature. 1994;369:340. 8183374 10.1038/369340a0

[12] Clynes R et al Modulation of immune complex-induced inflammation in vivo by the coordinate expression of activation and inhibitory Fc receptors. J Exp Med. 1999;189:179–185. 9874574 10.1084/jem.189.1.179 PMC1887693

[13] Espéli M et al FcγRIIb differentially regulates pre-immune and germinal center B cell tolerance in mouse and human. Nature Communications. 2019;10:1970. 10.1038/s41467-019-09434-0 PMC6488660 31036800

[14] Paul E , Nelde A, Verschoor A, Carroll MC. Follicular exclusion of autoreactive B cells requires FcγRIIb. International Immunology. 2007;19:365–373. 17307801 10.1093/intimm/dxm002

[15] Barlev AN et al FcγRIIB regulates autoantibody responses by limiting marginal zone B cell activation. J Clin Invest. 2022;132:e157250. 10.1172/JCI157250 PMC9435648 35819855

[16] Espéli M et al Analysis of a wild mouse promoter variant reveals a novel role for FcγRIIb in the control of the germinal center and autoimmunity. J Exp Med. 2012;209:2307–2319. 23109709 10.1084/jem.20121752 PMC3501356

[17] Li FB , Smith P, Ravetch JV. Inhibitory Fcγ receptor is required for the maintenance of tolerance through distinct mechanisms. J Immunol. 2014;192:3021–3028. 24563255 10.4049/jimmunol.1302934 PMC3967505

[18] Clynes R , Dumitru C, Ravetch JV. Uncoupling of immune complex formation and kidney damage in autoimmune glomerulonephritis. Science. 1998;279:1052–1054. 9461440 10.1126/science.279.5353.1052

[19] Beers SA et al Antigenic modulation limits the efficacy of anti-CD20 antibodies: implications for antibody selection. Blood. 2010;115:5191–5201. 20223920 10.1182/blood-2010-01-263533

[20] Clynes RA , Towers TL, Presta LG, Ravetch JV. Inhibitory Fc receptors modulate in vivo cytoxicity against tumor targets. Nat Med. 2000;6:443–446. 10742152 10.1038/74704

[21] Vargas FA , et al Lung TRACERx Consortium Fc-optimized anti-CD25 depletes tumor-infiltrating regulatory T cells and synergizes with PD-1 blockade to eradicate established tumors. Immunity. 2017;46:577–586. 28410988 10.1016/j.immuni.2017.03.013 PMC5437702

[22] Dahal LN et al STING activation reverses lymphoma-mediated resistance to antibody immunotherapy. Cancer Res. 2017;77:3619–3631. 28512240 10.1158/0008-5472.CAN-16-2784 PMC5500176

[23] Hussain K et al HIF activation enhances FcγRIIb expression on mononuclear phagocytes impeding tumor targeting antibody immunotherapy. J Exp Clin Cancer Res. 2022;41:131. 35392965 10.1186/s13046-022-02294-5 PMC8988350

[24] de Haij S et al Cytotoxicity of type I CD20 antibodies critically depends on Fc receptor ITAM signaling. Cancer Res. 2010;70:3209–3217. 20354182 10.1158/0008-5472.CAN-09-4109

[25] Lim SH et al Anti-CD20 monoclonal antibodies: historical and future perspectives. Haematol Hematol J. 2010;95:135–143. 10.3324/haematol.2008.001628 PMC2805725 19773256

[26] Minard-Colin V et al Lymphoma depletion during CD20 immunotherapy in mice is mediated by macrophage FcγRI, FcγRIII, and FcγRIV. Blood. 2008;112:1205–1213. 18495955 10.1182/blood-2008-01-135160 PMC2515149

[27] Otten MA et al Experimental antibody therapy of liver metastases reveals functional redundancy between FcγRI and FcγRIV. J Immunol. 2008;181:6829–6836. 18981101 10.4049/jimmunol.181.10.6829

[28] Simpson AP et al FcγRIIB controls antibody-mediated target cell depletion by ITIM-independent mechanisms. Cell Rep. 2022;40:111099. 35858562 10.1016/j.celrep.2022.111099 PMC9638011

[29] Daeron M et al The same tyrosine-based inhibition motif, in the intracytoplasmic domain of Fc-Gamma-Riib, regulates negatively Bcr-dependent, Tcr-dependent, and Fcr-dependent cell activation. Immunity. 1995;3:635–646. 7584153 10.1016/1074-7613(95)90134-5

[30] Dahan R et al FcγRs modulate the anti-tumor activity of antibodies targeting the PD-1/PD-L1 axis (vol 28, pg 285, 2015). Cancer Cell. 2015;28:543. 28854351 10.1016/j.ccell.2015.09.011

[31] Arlauckas SP et al In vivo imaging reveals a tumor-associated macrophage-mediated resistance pathway in anti-PD-1 therapy. Sci Transl Med. 2017;9:eaal3604. 10.1126/scitranslmed.aal3604 PMC5734617 28490665

[32] Knorr DA et al FcγRIIB is an immune checkpoint limiting the activity of treg-targeting antibodies in the tumor microenvironment. Cancer Immunol Res. 2024;12:322–333. 38147316 10.1158/2326-6066.CIR-23-0389 PMC10911703

[33] White AL et al Interaction with FcγRIIB is critical for the agonistic activity of anti-CD40 monoclonal antibody. J Immunol. 2011;187:1754–1763. 21742972 10.4049/jimmunol.1101135

[34] Li FB , Ravetch JV. Apoptotic and antitumor activity of death receptor antibodies require inhibitory Fcγ receptor engagement. P Natl Acad Sci USA. 2012;109:10966–10971. 10.1073/pnas.1208698109 PMC3390832 22723355

[35] Buchan SL et al Antibodies to costimulatory receptor 4-1BB enhance anti-tumor immunity via T regulatory cell depletion and promotion of CD8 T cell effector function. Immunity. 2018;49:958–970.e7. +. 30446386 10.1016/j.immuni.2018.09.014

[36] Liu LY et al Antibody-targeted TNFRSF activation for cancer immunotherapy: the role of FcγRIIB cross-linking. Front Pharmacol. 2022;13:924197. 35865955 10.3389/fphar.2022.924197 PMC9295861

[37] White AL et al Fcγ Receptor Dependency of Agonistic CD40 antibody in lymphoma therapy can be overcome through antibody multimerization. J Immunol. 2014;193:1828–1835. 25024386 10.4049/jimmunol.1303204

[38] Li FB , Ravetch JV. Inhibitory Fcγ receptor engagement drives adjuvant and anti-tumor activities of agonistic CD40 antibodies. Science. 2011;333:1030–1034. 21852502 10.1126/science.1206954 PMC3164589

[39] Mayes PA , Hance KW, Hoos A. The promise and challenges of immune agonist antibody development in cancer. Nat Rev Drug Discov. 2018;17:509–527. 29904196 10.1038/nrd.2018.75

[40] Beers SA , Glennie MJ, White AL. Influence of immunoglobulin isotype on therapeutic antibody function. Blood. 2016;127:1097–1101. 26764357 10.1182/blood-2015-09-625343 PMC4797141

[41] Bruggeman CW et al Tissue-specific expression of IgG receptors by human macrophages. PLoS One. 2019;14:e0223264. 31613876 10.1371/journal.pone.0223264 PMC6793881

[42] Hussain K et al Impact of human FcγR gene polymorphisms on IgG-triggered cytokine release: critical importance of cell assay format. Front Immunol. 2019;10:390. 30899264 10.3389/fimmu.2019.00390 PMC6417454

[43] Su KH et al Expression profile of FcγRIIB on leukocytes and its dysregulation in systemic lupus erythematosus. J Immunol. 2007;178:3272–3280. 17312177 10.4049/jimmunol.178.5.3272 PMC2824439

[44] Audia S et al Fcγ receptor expression on splenic macrophages in adult immune thrombocytopenia. Clin Exp Immunol. 2017;188:275–282. 28142207 10.1111/cei.12935 PMC5383444

[45] Nowicka M et al Prognostic significance of FCGR2B expression for the response of DLBCL patients to rituximab or obinutuzumab treatment. Blood Adv. 2021;5:2945–2957. 34323958 10.1182/bloodadvances.2021004770 PMC8361458

[46] Arthur SE et al Genome-wide discovery of somatic regulatory variants in diffuse large B-cell lymphoma. Nat Commun. 2018;9:4001. 30275490 10.1038/s41467-018-06354-3 PMC6167379

[47] Sun ZM et al FCGR2B as a prognostic and immune microenvironmental marker for gliomas based on transcriptomic analysis. Medicine. 2023;102:e35084. 37713871 10.1097/MD.0000000000035084 PMC10508392

[48] Roghanian A et al Cyclophosphamide enhances cancer antibody immunotherapy in the resistant bone marrow niche by modulating macrophage FcγR expression. Cancer Immunol Res. 2019;7:1876–1890. 31451483 10.1158/2326-6066.CIR-18-0835 PMC7780711

[49] Lee CS et al Expression of the inhibitory Fc gamma receptor IIB (FCGR2B, CD32B) on follicular lymphoma cells lowers the response rate to rituximab monotherapy (SAKK 35/98). Br J Haematol. 2015;168:145–148. 25142001 10.1111/bjh.13071

[50] Kyogoku C et al Fcγ receptor gene polymorphisms in Japanese patients with systemic lupus erythematosus: contribution of FCGR2B to genetic susceptibility. Arthritis and Rheumatism. 2002;46:1242–1254. 12115230 10.1002/art.10257

[51] Su KH et al A promoter haplotype of the immunoreceptor tyrosine-based inhibitory motif-bearing FcγRIIb alters receptor expression and associates with autoimmunity. II. Differential binding of GATA4 and Yin-Yang1 transcription factors and correlated receptor expression and function. J Immunol. 2004;172:7192–7199. 15153544 10.4049/jimmunol.172.11.7192

[52] Su KH et al A promoter haplotype of the immunoreceptor tyrosine-based inhibitory motif-bearing FcγRIIb alters receptor expression and associates with autoimmunity. I. regulatory FCGR2B polymorphisms and their association with systemic lupus erythematosus. J Immunol. 2004;172:7186–7191. 15153543 10.4049/jimmunol.172.11.7186

[53] Blank MC et al Decreased transcription of the human FCGR2B gene mediated by the -343 G/C promoter polymorphism and association with systemic lupus erythematosus. Human Genetics. 2005;117:220–227. 15895258 10.1007/s00439-005-1302-3

[54] Olferiev M , Masuda E, Tanaka S, Blank MC, Pricop L. The role of activating protein 1 in the transcriptional regulation of the human FCGR2B promoter mediated by the -343 G → C polymorphism associated with systemic lupus erythematosus. J Biol Chem. 2007;282:1738–1746. 17130130 10.1074/jbc.M605808200

[55] Xiu Y et al Transcriptional regulation of Fcgr2b gene by polymorphic promoter region and its contribution to humoral immune responses. J Immunol. 2002;169:4340–4346. 12370366 10.4049/jimmunol.169.8.4340

[56] Xu LL et al Through an ITIM-independent mechanism the FcγRIIB blocks B cell activation by disrupting the colocalized microclustering of the B cell receptor and CD19. J Immunol. 2014;192:5179–5191. 24790152 10.4049/jimmunol.1400101

[57] Isnardi I , Bruhns P, Bismuth G, Fridman WH, Daëron M. The SH2 domain-containing inositol 5-phosphatase SHIP1 is recruited to the intracytoplasmic domain of human FcγRIIB and is mandatory for negative regulation of B cell activation. Immunol Lett. 2006;104:156–165. 16406061 10.1016/j.imlet.2005.11.027

[58] Vaughan AT et al Inhibitory FcγRIIb (CD32b) becomes activated by therapeutic mAb in both cis and trans and drives internalization according to antibody specificity. Blood. 2014;123:669–677. 24227819 10.1182/blood-2013-04-490821

[59] Tipton TRW et al Antigenic modulation limits the effector cell mechanisms employed by type I anti-CD20 monoclonal antibodies. Blood. 2015;125:1901–1909. 25631769 10.1182/blood-2014-07-588376

[60] Lim SH et al Fc gamma receptor IIb on target B cells promotes rituximab internalization and reduces clinical efficacy. Blood. 2011;118:2530–2540. 21768293 10.1182/blood-2011-01-330357

[61] Vaughan AT et al Activatory and inhibitory Fcγ receptors augment rituximab-mediated internalization of CD20 independent of signaling via the cytoplasmic domain. J Biol Chem. 2015;290:5424–5437. 25568316 10.1074/jbc.M114.593806 PMC4342459

[62] Pritchard NR et al Autoimmune-prone mice share a promoter haplotype associated with reduced expression and function of the Fc receptor FcγRII. Curr Biol. 2000;10:227–230. 10704418 10.1016/s0960-9822(00)00344-4

[63] Berclaz PY , Shibata Y, Whitsett JA, Trapnell BC. GM-CSF, via PU.1, regulates alveolar macrophage FcγR-mediated phagocytosis and the IL-18/1FN-γ-mediated molecular connection between innate and adaptive immunity in the lung. Blood. 2002;100:4193–4200. 12393686 10.1182/blood-2002-04-1102

[64] Houston IB , Kamath MB, Schweitzer BL, Chlon TM, DeKoter RP. Reduction in PU.1 activity results in a block to B-cell development, abnormal myeloid proliferation, and neonatal lethality. Exp Hematol. 2007;35:1056–1068. 17588474 10.1016/j.exphem.2007.04.005 PMC1975786

[65] Willis SN et al Environmental sensing by mature B cells is controlled by the transcription factors PU.1 and SpiB. Nat Commun. 2017;8:1426. 29127283 10.1038/s41467-017-01605-1 PMC5681560

[66] Schweitzer BL et al Spi-C has opposing effects to PU.1 on gene expression in progenitor B cells. J Immunol. 2006;177:2195–2207. 16887979 10.4049/jimmunol.177.4.2195

[67] Roe JS , Mercan F, Rivera K, Pappin DJ, Vakoc CR. BET bromodomain inhibition suppresses the function of hematopoietic transcription factors in acute myeloid leukemia. Mol Cell. 2015;58:1028–1039. 25982114 10.1016/j.molcel.2015.04.011 PMC4475489

[68] Schweitzer BL , DeKoter RP. Analysis of gene expression and Ig transcription in PU.1/Spi-B-deficient progenitor B cell lines. J Immunol. 2004;172:144–154. 14688320 10.4049/jimmunol.172.1.144

[69] Solomon LA , Li SKH, Piskorz J, Xu LS, DeKoter RP. Genome-wide comparison of PU.1 and Spi-B binding sites in a mouse B lymphoma cell line. Cancer Res. 2015;75:2098. 10.1186/s12864-015-1303-0 PMC4334403 25765478

[70] Nishimura T et al Characterization of the human FcγRIIB gene promoter: human zinc-finger proteins (ZNF140 and ZNF91) that bind to different regions function as transcription repressors. Int Immunol. 2001;13:1075–1084. 11470777 10.1093/intimm/13.8.1075

[71] Cummin TEC et al BET inhibitors synergize with venetoclax to induce apoptosis in MYC-driven lymphomas with high BCL-2 expression. Blood Adv. 2020;4:3316–3328. 32717030 10.1182/bloodadvances.2020002231 PMC7391160

[72] Gearing LJ et al CiiiDER: A tool for predicting and analysing transcription factor binding sites. PLoS One. 2019;14:e0215495. 31483836 10.1371/journal.pone.0215495 PMC6726224

[73] Carter MJ et al BCR-signaling-induced cell death demonstrates dependency on multiple BH3-only proteins in a murine model of B-cell lymphoma. Cell Death Differ. 2016;23:303–312. 26184912 10.1038/cdd.2015.97 PMC4716310

[74] Grant CE , Bailey TL, Noble WS. FIMO: scanning for occurrences of a given motif. Bioinformatics. 2011;27:1017–1018. 21330290 10.1093/bioinformatics/btr064 PMC3065696

[75] Fornes O et al JASPAR 2020: update of the open-access database of transcription factor binding profiles. Nucleic Acids Res. 2020;48:D87–D92. 31701148 10.1093/nar/gkz1001 PMC7145627

[76] Karlsson M et al A single-cell type transcriptomics map of human tissues. Sci Adv. 2021;7:10.1126/sciadv.abh2169 PMC8318366 34321199

[77] Fernández JM , et al BLUEPRINT Consortium The BLUEPRINT data analysis portal. Cell Syst. 2016;3:491–495.e5.,. +. 27863955 10.1016/j.cels.2016.10.021 PMC5919098

[78] Gupta P , Gurudutta GU, Saluja D, Tripathi RP. PU.1 and partners: regulation of haematopoietic stem cell fate in normal and malignant haematopoiesis. J Cell Mol Med. 2009;13:4349–4363. 19382896 10.1111/j.1582-4934.2009.00757.x PMC4515051

[79] Yamamoto H , Kihara-Negishi F, Yamada T, Hashimoto Y, Oikawa T. Physical and functional interactions between the transcription factor PU.1 and the coactivator CBP. Oncogene. 1999;18:1495–1501. 10050886 10.1038/sj.onc.1202427

[80] Filippakopoulos P et al Selective inhibition of BET bromodomains. Nature. 2010;468:1067–1073. 20871596 10.1038/nature09504 PMC3010259

[81] Ozer HG et al BRD4 profiling identifies critical chronic lymphocytic leukemia oncogenic circuits and reveals sensitivity to PLX51107, a novel structurally distinct BET inhibitor. Cancer Discov. 2018;8:458–477. 29386193 10.1158/2159-8290.CD-17-0902 PMC5882533

[82] Boruchov AM et al Activating and inhibitory IgG Fc receptors on human DCs mediate opposing functions. J Clin Invest. 2005;115:2914–2923. 16167082 10.1172/JCI24772 PMC1201664

[83] de Waal Malefyt R et al Effects of IL-13 on phenotype, cytokine production, and cytotoxic function of human monocytes. Comparison with IL-4 and modulation by IFN-gamma or IL-10. J Immunol. 1993;151:6370–6381. 7902377

[84] Liu Y et al Cytokine-mediated regulation of activating and inhibitory Fcγ receptors in human monocytes. J Leukocyte Biol. 2005;77:767–776. 15703199 10.1189/jlb.0904532

[85] Cohen-Solal JFG et al Metastatic melanomas express inhibitory low affinity Fc Gamma receptor and escape humoral immunity. Dermatol Res Pract. 2010;2010:657406. 20672001 10.1155/2010/657406 PMC2905727

[86] Karlsson I et al Phase 1/2a clinical trial of BI-1206, a monoclonal antibody to Fc.riib, in. combination with rituximab in subjects with indolent B-cell non-hodgkin lymphoma that has relapsed or is refractory to rituximab. Blood. 2021;138:p1354.

[87] BioInvent. BioInvent presents positive first clinical data on anti-FcyRIIB antibody BI-1607 2023. Available from: https://www.bioinvent.com/en/press/bioinvent-presents-positive-first-clinical-data-anti-fcyriib-antibody-bi-1607-2181932.

[88] BioInvent. BioInvent Presents Promising Phase 1 Data for BI-1206 in Combination with KEYTRUDA^®^ (pembrolizumab) in Patients with Solid Tumors at ASCO 2024. 2024. https://www.bioinvent.com/en/press/bioinvent-presents-promising-phase-1-data-bi-1206-combination-keytrudar-pembrolizumab.

[89] Jiang VC et al Targeting FcγRIIB by antagonistic antibody BI-1206 improves the efficacy of rituximab-based therapies in aggressive mantle cell lymphoma. J Hematol Oncol. 2022;15:42. 35410313 10.1186/s13045-022-01257-9 PMC8996600

[90] Nutt SL , Metcalf D, D' Amico A, Polli M, Wu L. Dynamic regulation of PU.1 expression in multipotent hematopoietic progenitors. J Exp Med. 2005;201:221–231. 15657291 10.1084/jem.20041535 PMC2212785

[91] DeKoter RP , Singh H. Regulation of B lymphocyte and macrophage development by graded expression of PU.1. Science. 2000;288:1439–1441. 10827957 10.1126/science.288.5470.1439

[92] Dakic A , Wu L, Nutt SL. Is PU.1 a dosage-sensitive regulator of haemopoietic lineage commitment and leukaemogenesis? Trends Immunol. 2007;28:108–114. 17267285 10.1016/j.it.2007.01.006

[93] McKercher SR et al Targeted disruption of the PU.1 gene results in multiple hematopoietic abnormalities. Embo J. 1996;15:5647–5658. 8896458 PMC452309

[94] Scott EW , Simon MC, Anastasi J, Singh H. Requirement of transcription factor Pu.1 in the development of multiple hematopoietic lineages. Science. 1994;265:1573–1577. 8079170 10.1126/science.8079170

[95] Li GL , Hao WK, Hu WX. Transcription factor PU.1 and immune cell differentiation (Review). Int J Mol Med. 2020;46:1943–1950. 33125129 10.3892/ijmm.2020.4763

[96] Turkistany SA , DeKoter RP. The transcription factor PU.1 is a critical regulator of cellular communication in the immune system. Arch Immunol Ther Exp (Warsz). 2011;59:431–440. 21972017 10.1007/s00005-011-0147-9

[97] Barral A , Zaret KS. Pioneer factors: roles and their regulation in development. Trends Genet. 2024;40:134–148. 37940484 10.1016/j.tig.2023.10.007 PMC10873006

[98] van Riel B , Rosenbauer F. Epigenetic control of hematopoiesis: the PU.1 chromatin connection. Biol Chem. 2014;395:1265–1274. 25205721 10.1515/hsz-2014-0195

[99] Iwasaki H et al Distinctive and indispensable roles of PU.1 in maintenance of hematopoietic stem cells and their differentiation. Blood. 2005;106:1590–1600. 15914556 10.1182/blood-2005-03-0860 PMC1895212

[100] Polli M et al The development of functional B lymphocytes in conditional PU.1 knock-out mice. Blood. 2005;106:2083–2090. 15933053 10.1182/blood-2005-01-0283

[101] Garrett-Sinha LA et al PU.1 and Spi-B are required for normal B cell receptor-mediated signal transduction. Immunity. 1999;10:399–408. 10229183 10.1016/s1074-7613(00)80040-0

[102] Dahl R , Ramirez-Bergeron DL, Rao S, Simon MC. Spi-B can functionally replace PU.1 in myeloid but not lymphoid development. EMBO J. 2002;21:2220–2230. 11980719 10.1093/emboj/21.9.2220 PMC125373

[103] Schotte R et al The transcription factor Spi-B is expressed in plasmacytoid DC precursors and inhibits T-, B-, and NK-cell development. Blood. 2003;101:1015–1023. 12393575 10.1182/blood-2002-02-0438

[104] Zhao XH et al PU.1-c-Jun interaction is crucial for PU.1 function in myeloid development. Commun Biol. 2022;5:961. 36104445 10.1038/s42003-022-03888-7 PMC9474506

[105] Perez C , Coeffier E, Moreau-Gachelin F, Wietzerbin J, Benech PD. Involvement of the transcription factor Pu.1/Spi-1 in myeloid cell-restricted expression of an interferon-inducible gene encoding the human high-affinity Fc-Gamma receptor. Mol Cell Biol. 1994;14:5023–5031. 8035786 10.1128/mcb.14.8.5023-5031.1994 PMC359021

[106] Eichbaum QG , Iyer R, Raveh DP, Mathieu C, Ezekowitz RAB. Restriction of interferon-Gamma responsiveness and basal expression of the myeloid human Fc-Gamma-R1b gene is mediated by a functional Pu.1 site and a transcription initiator consensus. J Exp Med. 1994;179:1985–1996. 8195721 10.1084/jem.179.6.1985 PMC2191524

[107] Dahlqvist J et al Systematic identification of genomic elements that regulate expression and harbor variants linked with autoimmune disease. Hum Mol Genet. 2022;31:1946–1961. 34970970 10.1093/hmg/ddab372 PMC9239749

[108] Le Coz C et al Constrained chromatin accessibility in PU.1-mutated agammaglobulinemia patients. J Exp Med. 2021;218:e20201750. 10.1084/jem.20201750 PMC8105723 33951726

