# Supplementary material for: The ETS-family transcription factor PU.1 is a critical regulator of the inhibitory Fcγ receptor IIB expression in humans
Source: J Immunol. 2025 May 26;214(8):1937–50. doi: 10.1093/jimmun/vkaf109 (PMC12394989; doi:10.1093/jimmun/vkaf109)
Supplement: vkaf109_Supplementary_Data [file vkaf109_supplementary_data.pdf]

Supplementary Figures & Tables

Accompanying Carter et al.

The ETS-family transcription factor PU.1 is a critical regulator of the inhibitory Fcγ Receptor IIB expression in humans

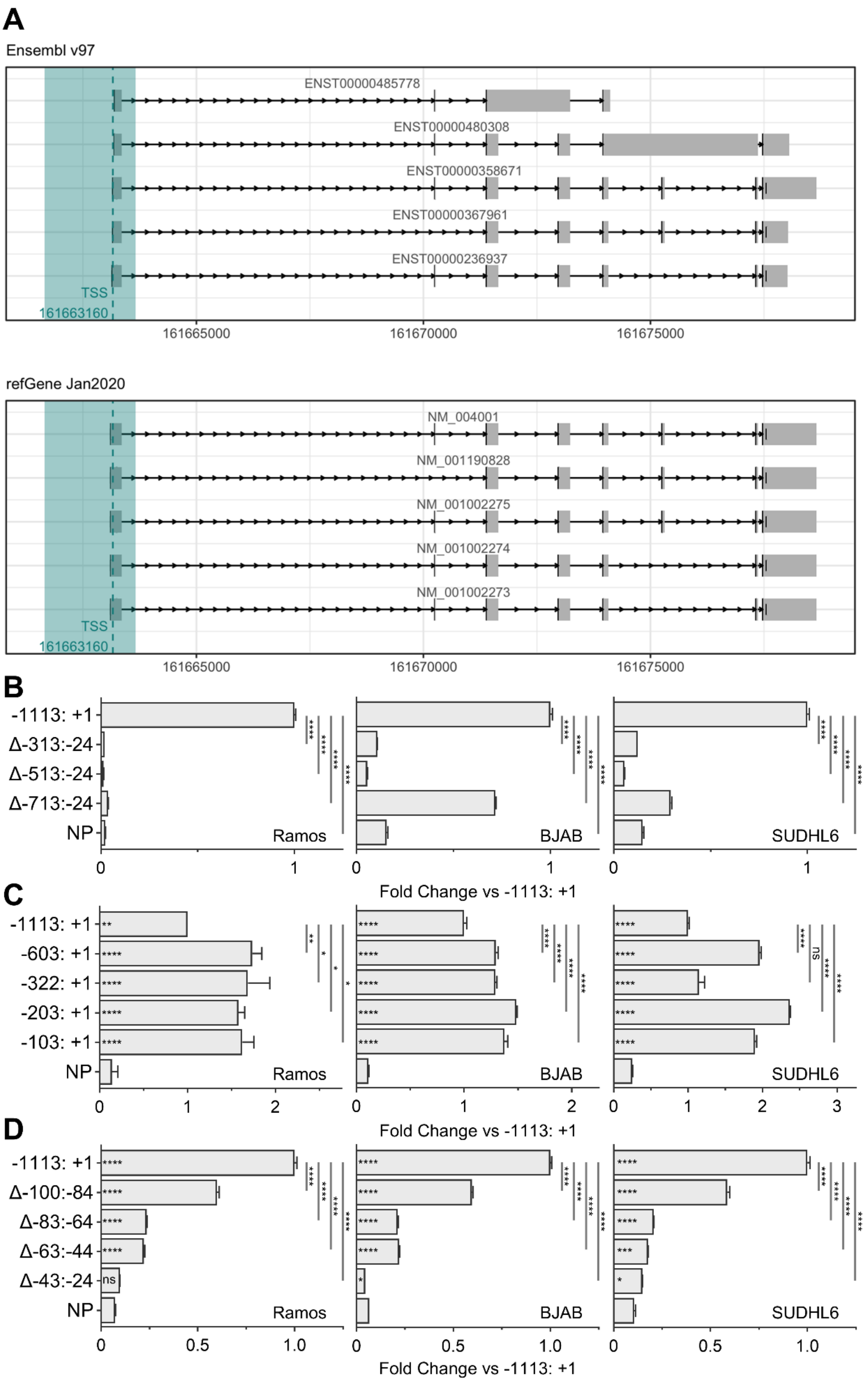

Supplementary Figure 1: FcγRIIB promoter activity is dictated by a TSS proximal 57 bp sequence in B-lymphoma cell lines. A) *FcγRIIB TSS was determined from the Ensembl v97 set of transcripts using CiiiDER TSS decision process and cross referenced with refGene 2020 transcripts. TSS is indicated as dashed line on gene model diagrams. Positional data represent co-ordinates using GRCh38.p12 (hg38) genome assembly and*

NC_000001.11 chromosome 1 reference. ***B-D)*** *Ramos, BJAB, and SUDHL6 B-lymphoma cell lines were transfected with the indicated FcγRIIB promoter reporter constructs (Supplementary Table 1 & Figure 1) and reporter activity assessed as described in Figure 1. Data represent mean, bars represent S.E.M. Statistical analyses were performed using one-way ANOVA adjusted for multiple comparisons using Tukey's test. ns = non-statistically significant, * = p <0.05, ** = p <0.005, *** = p <0.0005, ****=p <0.00005.*

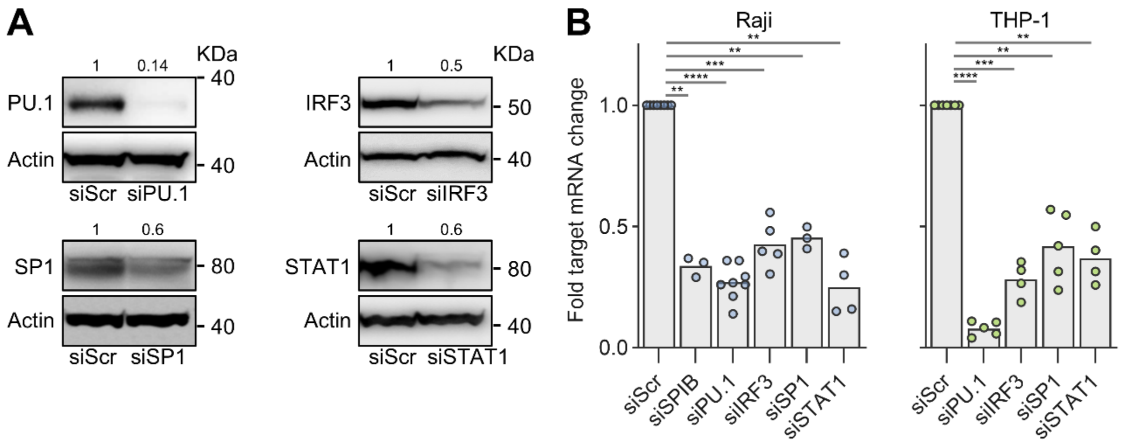

Supplementary Figure 2: siRNA-mediated gene silencing reduces target gene expression in Raji and THP-1 cell lines. A) *THP-1 cells were transfected with siRNA directed against PU.1, IRF3, SP1, or STAT1 or a scrambled siRNA control (denoted as siPU.1, siIRF3, siSP1, siSTAT1, or siScr, respectively) and the impact upon target gene protein levels assessed by immunoblot. Representative examples of at least 3 independent experiments are depicted.* ***B)*** *Raji or THP-1 cells were treated as in (**A**) and the impact of siRNA upon target gene mRNA levels assessed by qPCR. Data points represent independent experiments, each performed in triplicate, bars represent mean. Statistical analyses were performed using mixed model ANOVA corrected for multiple comparisons using Dunnett's test. ** = p <0.005, ****=p <0.00005.*

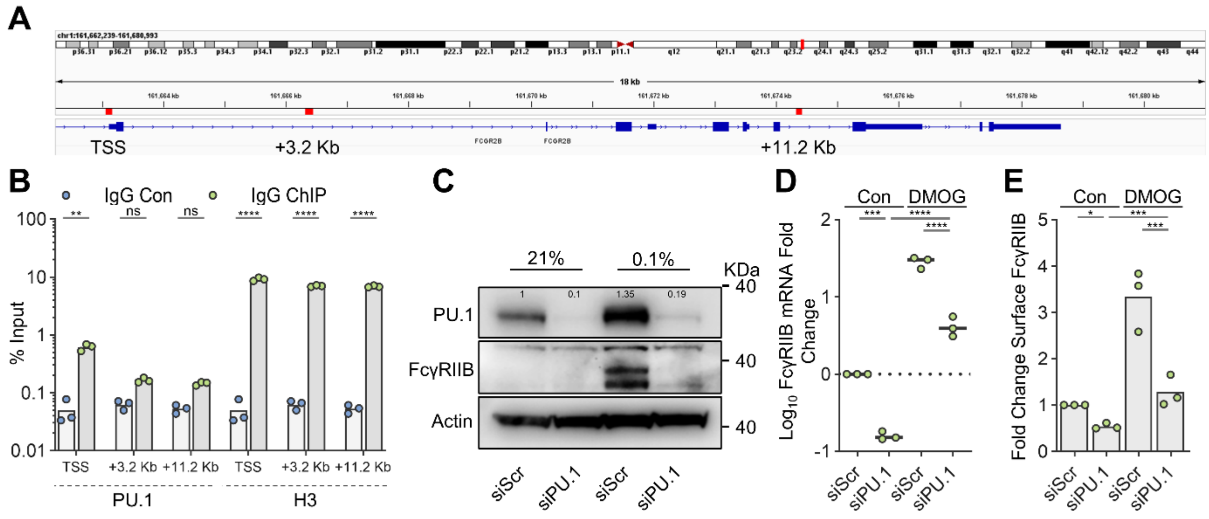

Supplementary Figure 3: PU.1 is critical for induced FcγRIIB gene expression. A-B) *Enrichment of PU.1 at FcγRIIB TSS proximal sequence was assessed by ChIP in Raji cells in comparison to intronic regions 3.2 and 11.2 kb downstream of TSS (primer-targeted regions highlighted in red in* ***A****). PU.1- or Histone H3-specific antibodies (IgG ChIP) were used in comparison to an appropriate isotype control (IgG Con). Data were normalised for primer amplification efficiency and expressed as % input. Data represents a representative example of 3 independent experiments, each performed in triplicate. Bars denote mean.* ***C)*** *THP-1 cells transfected with siPU.1 or siScr for 24 hours were cultured at either 21% or 0.1% O2 for a further 24 hours and assessed by immunoblot.* ***D-E)*** *THP-1 cells transfected with PU.1-targetting (siPU.1) or a scrambled siRNA (siScr) were treated with DMOG or an appropriate vehicle control (Con) as outlined in Figure 3E and FcγRIIB* ***D)*** *transcript or* ***E)*** *surface protein levels assessed by qPCR or flow cytometry, respectively. Data were normalised to siScr-transfected control-treated cells and expressed as fold change. Points represent independent experiments, each performed in triplicate. In all figures bars represent mean. Statistical analyses were performed using two-way (**B**) or one-way (**D, E**) ANOVA adjusted for multiple*

*comparisons using Sidak's test. ns = non-statistically significant, * = p <0.05, ** = p <0.005, *** = p <0.0005, ****=p <0.00005.*

A

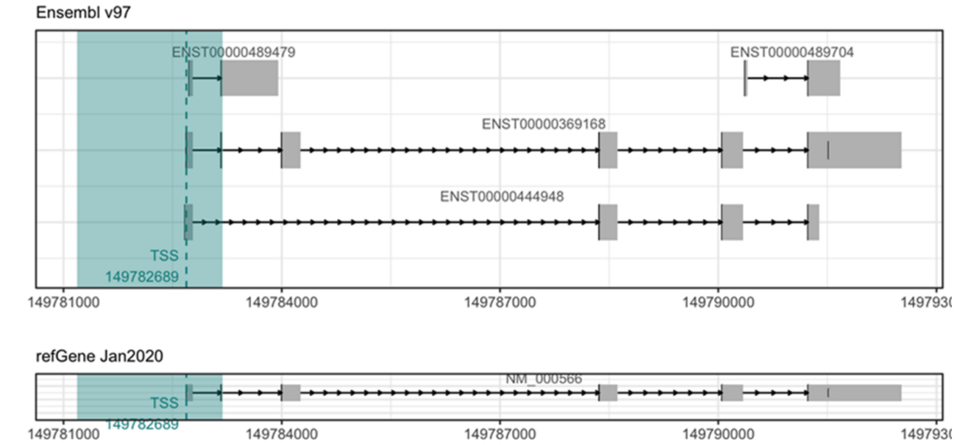

B

TF	Start	End	Strand	TFM	p.value
STAT1	-90	-79	-	MA0137.3	0.000000857
STAT3	-90	-79	-	MA0144.2	0.000000963
SPIC	-45	-31	-	MA0687.1	0.00000145
PU.1/SPI1	-50	-30	-	MA0080.5	0.00000294
IKZF1	-44	-32	-	MA1508.1	0.000016
SPIB	-48	-32	+	MA0081.2	0.0000424

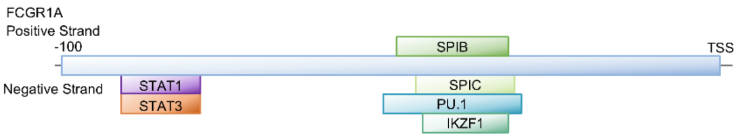

C

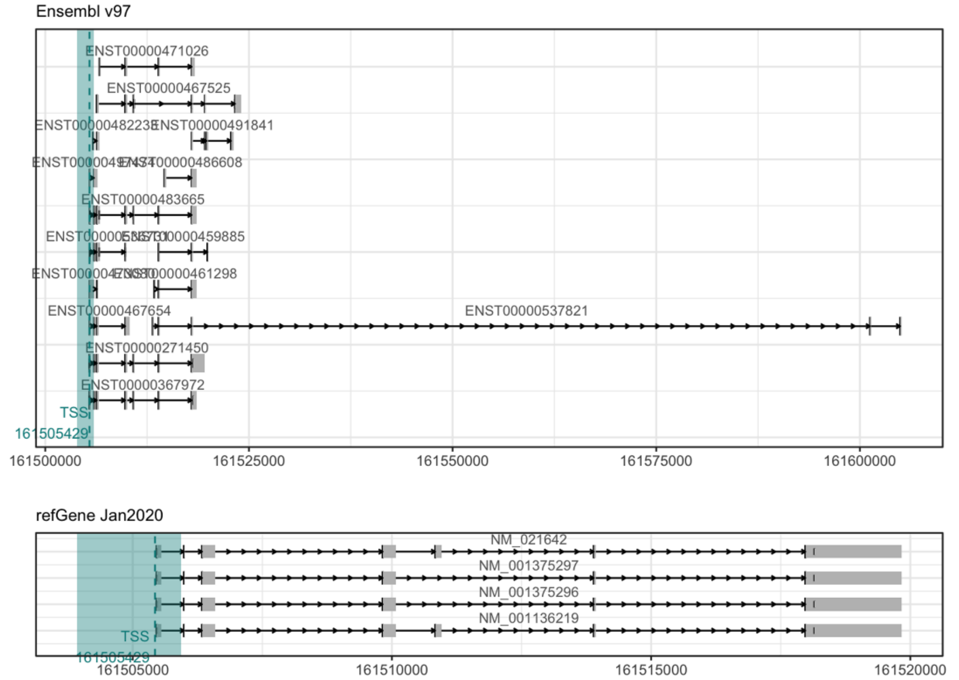

D

TF	Start	End	Strand	TFM	p.value
SPIC	-21	-7	-	MA0687.1	0.00000145
PU.1/SPI1	-26	-6	-	MA0080.5	0.00000223
NFYA	-85	-74	-	MA0060.3	0.00000266
NFYC	-85	-74	-	MA1644.1	0.00000495
REST	-61	-40	+	MA0138.2	0.0000139
IKZF1	-20	-8	-	MA1508.1	0.000016
PRDM4	-72	-61	-	MA1647.1	0.0000256
STAT1:STAT2	-37	-22	-	MA0517.1	0.0000388
SPIB	-24	-8	+	MA0081.2	0.0000424

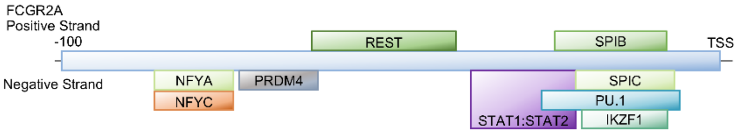

Supplementary Figure 4: TSS proximal PU.1 recognition motifs are conserved at FcγRI and FcγRIIA loci. A & C) *FcγRI & FcγRIIA TSS were defined as outlined for FcγRIIB in Supplementary Figure 1A.* ***B & D)*** *FcγRI & FcγRIIA loci were assessed for the presence of TF recognition motifs using the FIMO algorithm and filtered as described for FcγRIIB in Figure 2. Identified candidate TF motif positions are depicted in a to-scale representation of 0.1 kb TSS upstream regions of FCGR1A (**B**) and FCGR2A (**D**) loci.*

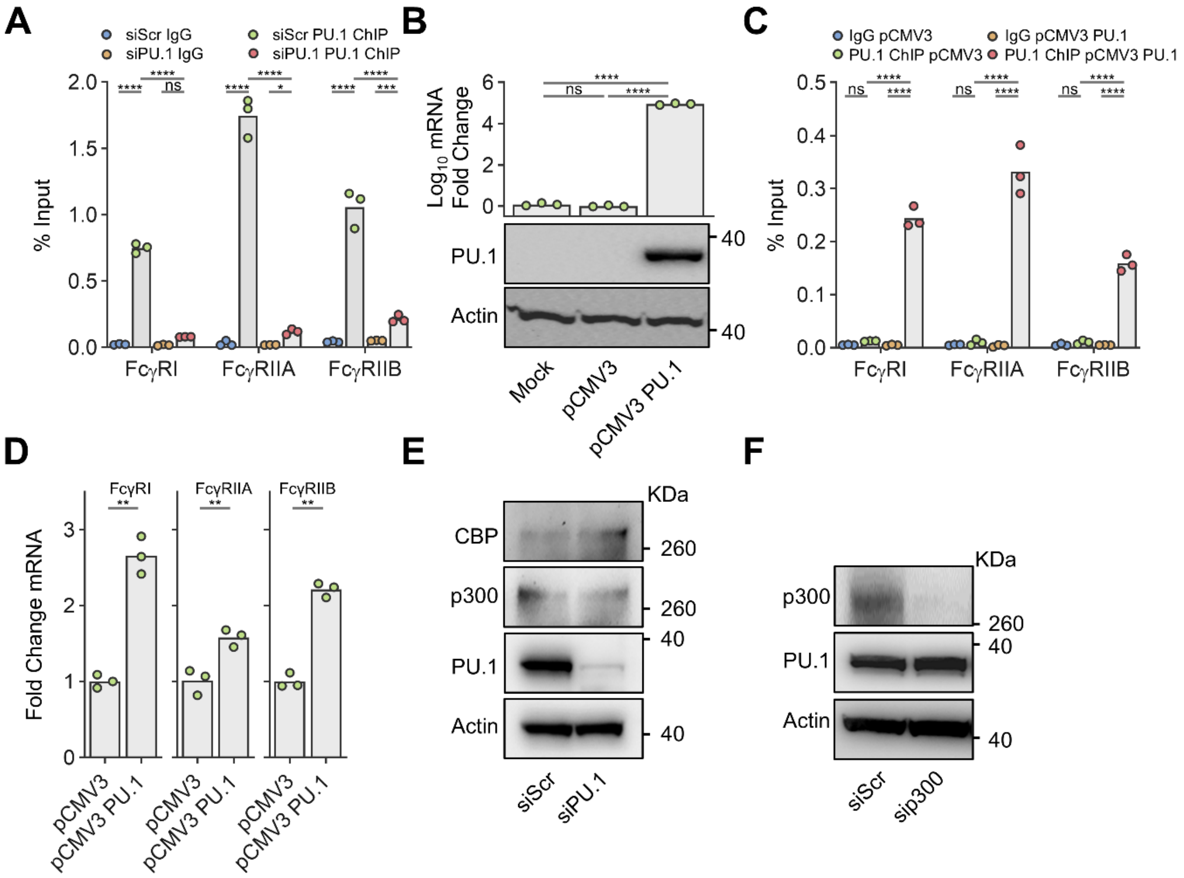

Supplementary Figure 5: Endogenous and exogenous PU.1 associates with TSS proximal locations of FcγR loci. A) *PU.1 association with TSS proximal sequence of FcγRI, FcγRIIA, and FcγRIIB loci was assessed by ChIP using PU.1 (PU.1 ChIP) antibody in comparison to an isotype control (IgG) in THP-1 cells transfected with PU.1-targetting siRNA (siPU.1) or a scrambled siRNA control (siScr). Data were normalised for primer amplification efficiency and expressed as % Input. Data depicts a representative example of 2 independent experiments each performed in triplicate.* ***B-D)*** *Exogenous PU.1 (pCMV3 PU.1), empty vector (pCMV3), or mock transfected HEK293F were assessed for PU.1 expression 72 hours post-transfection at the transcript and protein level by qPCR and immunoblot. Subsequently, PU.1 binding at FcγRI, FcγRIIA, and FcγRIIB TSS proximal sequence was assessed by (**C**) ChIP in comparison to an isotype control (IgG) as in (**A**) and impacts upon FcγR transcript expression assessed by (**D**) qPCR. Data represent triplicates.* ***E-F)*** *THP1-cells were transfected with either (**E**) PU.1- or (**F**) p300-targetting siRNA (siPU.1, sip300) in comparison to a scrambled siRNA control (siScr) and assessed by immunoblot. Statistical analyses were performed using 2-way (**A, C**) or one-way (**B**) ANOVA adjusted for multiple comparisons using Tukey's or Sidak's test where appropriate. Comparisons in (**D**) were made using paired T-test analysis. ns = non-statistically significant, * = $p < 0.05$, ** = $p < 0.005$, *** = $p < 0.0005$, ****=$p < 0.00005$. Bars in all figures represent mean.*

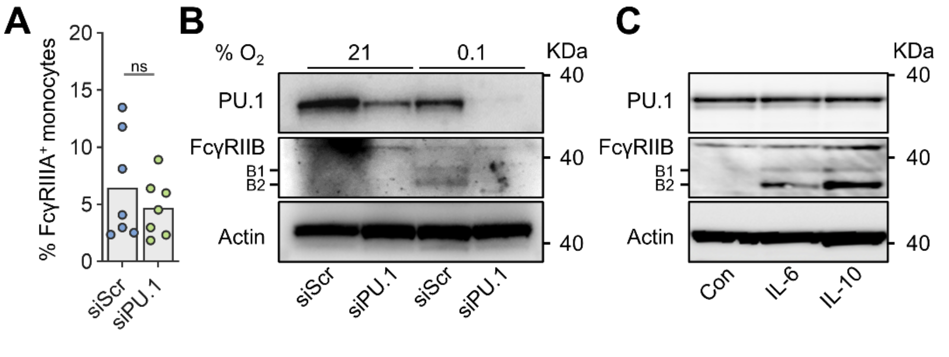

Supplementary Figure 6: Loss of PU.1 expression does not impact the frequency of FcγRIIIA$^+$ monocytes. A) *Purified primary human monocytes were transfected with a PU.1-targetting (siPU.1) or scrambled control siRNA*

(siScr) for 24 hours and the relative frequency of FcγRIIIA+ monocytes assessed by flow cytometry. Dots represent individual donors. Statistical analysis was performed using paired Student's t-test analysis, n.s. = non-statistically significant. ***B)*** *Purified primary human monocytes obtained from healthy donor PBMC were transfected with a PU.1-targetting (siPU.1) or scrambled control siRNA (siScr) for 24 hours prior to culture at 21% or 0.1% O2 for a further 24 hours. PU.1 and FcγRIIB expression were assessed by immunoblot.* ***C)*** *Primary human monocytes treated with 50 ng/mL IL-6, 50 ng/mL IL-10, or a vehicle control (Con) for 24 hours were assessed by immunoblot analysis.*

Insert	Sequence
-1113: +1	Chr1:161662047:GAGGATGATGACAGAACGCAAGAAAAGAGAACTAGCATTCACCGGACACCCACGATGTACCAAGCACTTTGCTAACCCTCATATTCTCTTTTACTTTCCAAAAACCTGTAGTACTGTGGTTCTCAGCCAGGGGCAATTCATCCCCTGGGGAACACTTGCTAATATCTGGGGGCATATTTGTTTTCACAACTGGGAGTGCCACTGGCATCTAACAGGTAGAGCCCAGGAGTGCTGCGGAACATCCTACAATGCTCAGGGAAGGTCCTCACAAGAATAATTTGGCCCACAATGTCCATAGTGCTCAGGCTGAGAAACCGTGCTTAAATGGTAGGCACAATAATCTTCACTTTTACAGATTGGAACCTGACACTCTGAGAAGCCACCTGGCATTCAACCCGAAACCTAACACAGCTCCAAAGCCCATGCTCTTTCACCATGCCGTTGCAGTGAGAACAGGGATGGAAATGAGGGTGGCAAAAATGACCAAGATACAAAACCAGGGCACAGATTGGTGCTCAATAGATACTTATTGGGATATTCATTAAATAGAAGAATGAATAAGAAAAAGAATGAATGAGGGCAGGGGAATAATGAGGATGAGTGTGGTCATTCTATTGCCATCCTGACATACCTCCTTGTCCTTGTTCCACAACTCAGCAGTGAGTCTGGGATTATGACAATAGAGAAAATTAAATGATGGTAGGTGGCCTGGAGTCCCCATGCTCAATTTCAAGAAGCATCCAGATTCCAGGGCCTGGGTCTCCAAATGGAAGTAGAAGTACTAGAAGATTGCTGGTGCACGCTGTCCTGCATCACCCTTTCTCAGGAGGATAGAGACTGAAACAGGAGGTTCTGAGCTGAGTTTTGGTGACCATTTCCCTCTTTCTCCCAGAGGCCCAGGCCAGCTGTGGCCTCAGAGGAAGAAGAAGGGAGTTGTTTCCCTAGTTTCTAAAATTTCTGTGAATTTGAACATGGGCTACACCAGATTTATTCTGGGAAGCTCTGAATCTTCTAGGAGGGAAAGACTGAGAGGAAAGAGGGTGGAAAGGGAGGAGCCTGTGATAAAACAGAACATTTCTTTTTCACTTCCCCTTTCAGACTCCAGAATTTGTTT:Chr1:161663160
-603: +1	Chr1:161662557:GGTGCTCAATAGATACTTATTGGGATATTCATTAAATAGAAGAATGAATAAGAAAAAGAATGAATGAGGGCAGGGGAATAATGAGGATGAGTGTGGTCATTCTATTGCCATCCTGACATACCTCCTTGTCCTTGTTCCACAACTCAGCAGTGAGTCTGGGATTATGACAATAGAGAAAATTAAATGATGGTAGGTGGCCTGGAGTCCCCATGCTCAATTTCAAGAAGCATCCAGATTCCAGGGCCTGGGTCTCCAAATGGAAGTAGAAGTACTAGAAGATTGCTGGTGCACGCTGTCCTGCATCACCCTTTCTCAGGAGGATAGAGACTGAAACAGGAGGTTCTGAGCTGAGTTTTGGTGACCATTTCCCTCTTTCTCCCAGAGGCCCAGGCCAGCTGTGGCCTCAGAGGAAGAAGAAGGGAGTTGTTTCCCTAGTTTCTAAAATTTCTGTGAATTTGAACATGGGCTACACCAGATTTATTCTGGGAAGCTCTGAATCTTCTAGGAGGGAAAGACTGAGAGGAAAGAGGGTGGAAAGGGAGGAGCCTGTGATAAAACAGAACATTTCTTTTTCACTTCCCCTTTCAGACTCCAGAATTTGTTT:Chr1:161663160
-322: +1	Chr1:161662838:GCTGGTGCACGCTGTCCTGCATCACCCTTTCTCAGGAGGATAGAGACTGAAACAGGAGGTTCTGAGCTGAGTTTTGGTGACCATTTCCCTCTTTCTCCCAGAGGCCCAGGCCAGCTGTGGCCTCAGAGGAAGAAGAAGGGAGTTGTTTCCCTAGTTTCTAAAATTTCTGTGAATTTGAACATGGGCTACACCAGATTTATTCTGGGAAGCTCTGAATCTTCTAGGAGGGAAAGACTGAGAGGAAAGAGGGTGGAAAGGGAGGAGCCTGTGATAAAACAGAACATTTCTTTTTCACTTCCCCTTTCAGACTCCAGAATTTGTTT:Chr1:161663160
203: +1	Chr1:161662957:GCCTCAGAGGAAGAAGAAGGGAGTTGTTTCCCTAGTTTCTAAAATTTCTGTGAATTTGAACATGGGCTACACCAGATTTATTCTGGGAAGCTCTGAATCTTCTAGGAGGGAAAGACTGAGAGGAAAGAGGGTGGAAAGGGAGGAGCCTGTGATAAAACAGAACATTTCTTTTTCACTTCCCCTTTCAGACTCCAGAATTTGTTT:Chr1:161663160
-103: +1	Chr1:161663057:TCTAGGAGGGAAAGACTGAGAGGAAAGAGGGTGGAAAGGGAGGAGCCTGTGATAAAACAGAACATTTCTTTTTCACTTCCCCTTTCAGACTCCAGAATTTGTTT:Chr1:161663160
Δ-313 : -24	Chr1:161662047:GAGGATGATGACAGAACGCAAGAAAAGAGAACTAGCATTCACCGGACACCCACGATGTACCAAGCACTTTGCTAACCCTCATATTCTCTTTTACTTTCCAAAAACCTGTAGTACTGTGGTTCTCAGCCAGGGGCAATTCATCCCCTGGGGAACACTTGCTAATATCTGGGGGCATATTTGTTTTCACAACTGGGAGTGCCACTGGCATCTAACAGGTAGAGCCCAGGAGTGCTGCGGAACATCCTACAATGCTCAGGGAAGGTCCTCACAAGAATAATTTGGCCCACAATGTCCATAGTGCTCAGGCTGAGAAACCGTGCTTAAATGGTAGGCACAATAATCTTCACTTTTACAGATTGGAACCTGACACTCTGAGAAGCCACCTGGCATTCAACCCGAAACCTAACACAGCTCCAAAGCCCATGCTCTTTCACCATGCCGTTGCAGTGAGAACAGGGATGGAAATGAGGGTGGCAAAAATGACCAAGATACAAAACCAGGGCACAGATTGGTGCTCAATAGATACTTATTGGGATATTCATTAAATAGAAGAATGAATAAGAAAAAGAATGAATGAGGGCAGGGGAATAATGAGGATGAGTGTGGTCATTCTATTGCCATCCTGACATACCTCCTTGTCCTTGTTCCACAACTCAGCAGTGAGTCTGGGATTATGACAATAGAGAAAATTAAATGATGGTAGGTGGCCTGGAGTCCCCATGCTCAATTTCAAGAAGCATCCAGATTCCAGGGCCTGGGTCTCCAAATGGAAGTAGAAGTACTAGAAGATTGCTGGTGCA:Chr1:161662846**TSSProximal24bp**Chr1:161663137:CCTTTCAGACTCCAGAATTTGTTT:Chr1:161663160
Δ-513 : -24	Chr1:161662047:GAGGATGATGACAGAACGCAAGAAAAGAGAACTAGCATTCACCGGACACCCACGATGTACCAAGCACTTTGCTAACCCTCATATTCTCTTTTACTTTCCAAAAACCTGTAGTACTGTGGTTCTCAGCCAGGGGCAATTCATCCCCTGGGGAACACTTGCTAATATCTGGGGGCATATTTGTTTTCACAACTGGGAGTGCCACTGGCATCTAACAGGTAGAGCCCAGGAGTGCTGCGGAACATCCTACAATGCTCAGGGAAGGTCCTCACAAGAATAATTTGGCCCACAATGTCCATAGTGCTCAGGCTGAGAAACCGTGCTTAAATGGTAGGCACAATAATCTTCACTTTTACAGATTGGAACCTGACACTCTGAGAAGCCACCTGGCATTCAACCCGAAACCTAACACAGCTCCAAAGCCCATGCTCTTTCACCATGCCGTTGCAGTGAGAACAGGGATGGAAATGAGGGTGGCAAAAATGACCAAGATACAAAACCAGGGCACAGATTGGTGCTCAATAGATACTTATTGGGATATTCATTAAATAGAAGAATGAATAAGAAAAAGAATGAATGAGGGCAGGGGAATAATGAGGATGA:Chr1:161662646**TSSProximal24bp**Chr1:161663137:CCTTTCAGACTCCAGAATTTGTTT:Chr1:161663160
Δ-713 : -24	Chr1:161662047:GAGGATGATGACAGAACGCAAGAAAAGAGAACTAGCATTCACCGGACACCCACGATGTACCAAGCACTTTGCTAACCCTCATATTCTCTTTTACTTTCCAAAAACCTGTAGTACTGTGGTTCTCAGCCAGGGGCAATTCATCCCCTGGGGAACACTTGCTAATATCTGGGGGCATATTTGTTTTCACAACTGGGAGTGCCACTGGCATCTAACAGGTAGAGCCCAGGAGTGCTGCGGAACATCCTACAATGCTCAGGGAAGGTCCTCACAAGAATAATTTGGCCCACAATGTCCATAGTGCTCAGGCTGAGAAACCGTGCTTAAATGGTAGGCACAATAATCTTCACTTTTACAGATTGGAACCTGACACTCTGAGAAGCCACCTGGCATTCAACCCGAA:Chr1:161662446**TSSProximal24bp**Chr1:161663137: CCTTTCAGACTCCAGAATTTGTTT:Chr1:161663160
Δ-103: -84	Chr1:161662160GAGGATGATGACAGAACGCAAGAAAAGAGAACTAGCATTCACCGGACACCCACGATGTACCAAGCACTTTGCTAACCCTCATATTCTCTTTTACTTTCCAAAAACCTGTAGTACTGTGGTTCTCAGCCAGGGGCAATTCATCCCCTGGGGAACACTTGCTAATATCTGGGGGCATATTTGTTTTCACAACTGGGAGTGCCACTGGCATCTAACAGGTAGAGCCCAGGAGTGCTGCGGAACATCCTACAATGCTCAGGGAAGGTCCTCACAAGAATAATTTGGCCCACAATGTCCATAGTGCTCAGGCTGAGAAACCGTGCTTAAATGGTAGGCACAATAATCTTCACTTTTACAGATTGGAACCTGACACTCTGAGAAGCCACCTGGCATTCAACCCGAAACCTAACACAGCTCCAAAGCCCATGCTCTTTCACCATGCCGTTGCAGTGAGAACAGGGATGGAAATGAGGGTGGCAAAAATGACCAAGATACAAAACCAGGGCACAGATTGGTGCTCAATAGATACTTATTGGGATATTCATTAAATAGAAGAATGAATAAGAAAAAGAATGAATGAGGGCAGGGGAATAATGAGGATGAGTGTGGTCATTCTATTGCCATCCTGACATACCTCCTTGTCCTTGTTCCACAACTCAGCAGTGAGTCTGGGATTATGACAATAGAGAAAATTAAATGATGGTAGGTGGCCTGGAGTCCCCATGCTCAATTTCAAGAAGCATCCAGATTCCAGGGCCTGGGTCTCCAAATGGAAGTAGAAGTACTAGAAGATTGCTGGTGCACGCTGTCCTGCATCACCCTTTCTCAGGAGGATAGAGACTGAAACAGGAGGTTCTGAGCTGAGTTTTGGTGACCATTTCCCTCTTTCTCCCAGAGGCCCAGGCCAGCTGTGGCCTCAGAGGAAGAAGAAGGGAGTTGTTTCCCTAGTTTCTAAAATTTCTGTGAATTTGAACATGGGCTACACCAGATTTATTCTGGGAAGCTCTGAATCTChr1:161663056 Chr1:161663077AGGAAAGAGGGTGGAAAGGGAGGAGCCTGTGATAAAACAGAACATTTCTTTTTCACTTCCCCTTTCAGACTCCAGAATTTGTTTChr1:161663160

Δ-83: -64	*Chr1:161662160GAGGATGATGACAGAACGCAAGAAAAGAGAACTAGCATTCACCGGACACCCACGATGTACCAAGCACTTTGCTAACCCTCATATTCTCTTTTACTTTCCAAAAACCTGTAGTACTGTGGTTCTCAGCCAGGGGCAATTCATCCCCTGGGGAACACTTGCTAATATCTGGGGGCATATTTGTTTTCACAACTGGGAGTGCCACTGGCATCTAACAGGTAGAGCCCAGGAGTGCTGCGGAACATCCTACAATGCTCAGGGAAGGTCCTCACAAGAATAATTTGGCCCACAATGTCCATAGTGCTCAGGCTGAGAAACCGTGCTTAAATGGTAGGCACAATAATCTTCACTTTTACAGATTGGAACCTGACACTCTGAGAAGCCACCTGGCATTCAACCCGAAACCTAACACAGCTCCAAAGCCCATGCTCTTTCACCATGCCGTTGCAGTGAGAACAGGGATGGAAATGAGGGTGGCAAAAATGACCAAGATACAAAACCAGGGCACAGATTGGTGCTCAATAGATACTTATTGGGATATTCATTAAATAGAAGAATGAATAAGAAAAAGAATGAATGAGGGCAGGGGAATAATGAGGATGAGTGTGGTCATTCTATTGCCATCCTGACATACCTCCTTGTCCTTGTTCCACAACTCAGCAGTGAGTCTGGGATTATGACAATAGAGAAAATTAAATGATGGTAGGTGGCCTGGAGTCCCCATGCTCAATTTCAAGAAGCATCCAGATTCCAGGGCCTGGGTCTCCAAATGGAAGTAGAAGTACTAGAAGATTGCTGGTGCACGCTGTCCTGCATCACCCTTTCTCAGGAGGATAGAGACTGAAACAGGAGGTTCTGAGCTGAGTTTTGGTGACCATTTCCCTCTTTCTCCCAGAGGCCCAGGCCAGCTGTGGCCTCAGAGGAAGAAGAAGGGAGTTGTTTCCCTAGTTTCTAAAATTTCTGTGAATTTGAACATGGGCTACACCAGATTTATTCTGGGAAGCTCTGAATCTTCTAGGAGGGAAAGACTGAGChr1:161663076 Chr1:161663097AGGAGCCTGTGATAAAACAGAACATTTCTTTTTCACTTCCCCTTTCAGACTCCAGAATTTGTTTChr1:161663160*
Δ-63: -44	*Chr1:161662160GAGGATGATGACAGAACGCAAGAAAAGAGAACTAGCATTCACCGGACACCCACGATGTACCAAGCACTTTGCTAACCCTCATATTCTCTTTTACTTTCCAAAAACCTGTAGTACTGTGGTTCTCAGCCAGGGGCAATTCATCCCCTGGGGAACACTTGCTAATATCTGGGGGCATATTTGTTTTCACAACTGGGAGTGCCACTGGCATCTAACAGGTAGAGCCCAGGAGTGCTGCGGAACATCCTACAATGCTCAGGGAAGGTCCTCACAAGAATAATTTGGCCCACAATGTCCATAGTGCTCAGGCTGAGAAACCGTGCTTAAATGGTAGGCACAATAATCTTCACTTTTACAGATTGGAACCTGACACTCTGAGAAGCCACCTGGCATTCAACCCGAAACCTAACACAGCTCCAAAGCCCATGCTCTTTCACCATGCCGTTGCAGTGAGAACAGGGATGGAAATGAGGGTGGCAAAAATGACCAAGATACAAAACCAGGGCACAGATTGGTGCTCAATAGATACTTATTGGGATATTCATTAAATAGAAGAATGAATAAGAAAAAGAATGAATGAGGGCAGGGGAATAATGAGGATGAGTGTGGTCATTCTATTGCCATCCTGACATACCTCCTTGTCCTTGTTCCACAACTCAGCAGTGAGTCTGGGATTATGACAATAGAGAAAATTAAATGATGGTAGGTGGCCTGGAGTCCCCATGCTCAATTTCAAGAAGCATCCAGATTCCAGGGCCTGGGTCTCCAAATGGAAGTAGAAGTACTAGAAGATTGCTGGTGCACGCTGTCCTGCATCACCCTTTCTCAGGAGGATAGAGACTGAAACAGGAGGTTCTGAGCTGAGTTTTGGTGACCATTTCCCTCTTTCTCCCAGAGGCCCAGGCCAGCTGTGGCCTCAGAGGAAGAAGAAGGGAGTTGTTTCCCTAGTTTCTAAAATTTCTGTGAATTTGAACATGGGCTACACCAGATTTATTCTGGGAAGCTCTGAATCTTCTAGGAGGGAAAGACTGAGAGGAAAGAGGGTGGAAAGGG Chr1:161663096 Chr1:161663117AACATTTCTTTTTCACTTCCCCTTTCAGACTCCAGAATTTGTTTChr1:161663160*
Δ-43: -44	*Chr1:161662160GAGGATGATGACAGAACGCAAGAAAAGAGAACTAGCATTCACCGGACACCCACGATGTACCAAGCACTTTGCTAACCCTCATATTCTCTTTTACTTTCCAAAAACCTGTAGTACTGTGGTTCTCAGCCAGGGGCAATTCATCCCCTGGGGAACACTTGCTAATATCTGGGGGCATATTTGTTTTCACAACTGGGAGTGCCACTGGCATCTAACAGGTAGAGCCCAGGAGTGCTGCGGAACATCCTACAATGCTCAGGGAAGGTCCTCACAAGAATAATTTGGCCCACAATGTCCATAGTGCTCAGGCTGAGAAACCGTGCTTAAATGGTAGGCACAATAATCTTCACTTTTACAGATTGGAACCTGACACTCTGAGAAGCCACCTGGCATTCAACCCGAAACCTAACACAGCTCCAAAGCCCATGCTCTTTCACCATGCCGTTGCAGTGAGAACAGGGATGGAAATGAGGGTGGCAAAAATGACCAAGATACAAAACCAGGGCACAGATTGGTGCTCAATAGATACTTATTGGGATATTCATTAAATAGAAGAATGAATAAGAAAAAGAATGAATGAGGGCAGGGGAATAATGAGGATGAGTGTGGTCATTCTATTGCCATCCTGACATACCTCCTTGTCCTTGTTCCACAACTCAGCAGTGAGTCTGGGATTATGACAATAGAGAAAATTAAATGATGGTAGGTGGCCTGGAGTCCCCATGCTCAATTTCAAGAAGCATCCAGATTCCAGGGCCTGGGTCTCCAAATGGAAGTAGAAGTACTAGAAGATTGCTGGTGCACGCTGTCCTGCATCACCCTTTCTCAGGAGGATAGAGACTGAAACAGGAGGTTCTGAGCTGAGTTTTGGTGACCATTTCCCTCTTTCTCCCAGAGGCCCAGGCCAGCTGTGGCCTCAGAGGAAGAAGAAGGGAGTTGTTTCCCTAGTTTCTAAAATTTCTGTGAATTTGAACATGGGCTACACCAGATTTATTCTGGGAAGCTCTGAATCTTCTAGGAGGGAAAGACTGAGAGGAAAGAGGGTGGAAAGGGAGGAGCCTGTGATAAAACAGChr1: 161663116 Chr1: 161663137CCTTTCAGACTCCAGAATTTGTTTChr1: 161663160*

Supplementary Table 1: FcγRIIB promoter reporter construct sequences. *Sequence information is demonstrated for all FcγRIIB reporter constructs utilised in this study. Sequence is preceded by chromosome 1 co-ordinates using the NC_000001.11 chromosome 1 reference sequence.*

siRNA Target	Manufacturer	Item #
SPIB	Dharmacon	SMARTPool L-019722-00
PU.1	Invitrogen	Stealth siRNA HSS186060
IRF3	Invitrogen	Stealth siRNA HSS105507
SP1	Dharmacon	SMARTPool L-026959-00
STAT1	Dharmacon	SMARTPool L-003543-00
Scram 1	Dharmacon	SMARTPool D-001810-10
Scram 2	Invitrogen	Stealth siRNA Negative Control, Med GC 12935300

Supplementary Table 2: Gene-specific siRNA utilised for gene knockdown. *Product numbers and manufacturers of gene-specific siRNA utilised in this study.*

ChIP mAb Target	Manufacturer	Item #	Quantity per IP (μg)
SPIB	Cell Signalling Technology	14337	1
PU.1	Cell Signalling Technology	2266	0.4
IRF1	Cell Signalling Technology	8478	0.66
ZNF263	Abnova	H00010127-A01	1
IRF3	Cell Signalling Technology	4302	1
ETV6	Sigma-Aldrich	HPA000264	1
SP1	Cell Signalling Technology	9389	1
STAT1	Cell Signalling Technology	14994	1
STAT2	Cell Signalling Technology	72604	0.77
Histone H3	Cell Signalling Technology	4620	2.7
CBP	Cell Signalling Technology	7389	1
p300	Cell Signalling Technology	54062	1
BRD4	Cell Signalling Technology	13440	1
Negative Control	Cell Signalling Technology	3900	As appropriate

Supplementary Table 3: Western Blot and ChIP Antibodies. *Details of antibodies utilised for chromatin immunoprecipitation analysis in this study.*

Primer Target	Sequence	Product Chromosome 1 Co-ordinates (NC_000001.11 RefSeq)
FcγRIIB TSS	Fwd: TCTAGGAGGGAAAGACTGAGAGG<br>Rev: CCCTAGAGGGCAAACAAATTCTGG	161663057 - 161663171
FcγRIIB TSS + 3.2 Kb	Fwd: CTATGTAGCTGGGTGGATAACGAAG<br>Rev: CCCTCAGCAGTCCACTAAAACT	161666311 - 161666445
FcγRIIB TSS + 11.2 Kb	Fwd: TCAGCATTGTGAGGAAGACAGG<br>Rev: GAGCCTCCTCTGTACTTCTATCCTA	161674328 - 161674423
FcγRIABCp TSS	Fwd: CCACCAGCAGAACCTCTTCAATA<br>Rev: GAGTCCAACTTACCCCAAAGGAG	FcγRIA: 149782675 – 149782787<br>FcγRIB: 121087326 – 121087416<br>FcγRICp:143874724 – 143874836
FcγRIIA TSS	Fwd: ACCATTTCCTTCCTCTTTTCTAAGC<br>Rev: ACATTTGGGTCTCCATAGTCATCC	161505403 – 161505489

Supplementary Table 4: Primer sequences utilised in Chromatin Immunoprecipitation. *Primer sequences and the predicted co-ordinates of amplified regions of the NC_000001.11 chromosome 1 reference sequence utilised in chromatin immunoprecipitation qPCR analysis.*

Taqman Assay Target	Assay ID
SPIB	Hs00162150_m1
PU.1	Hs02786711_m1
IRF3	Hs01547283_m1
SP1	Hs00916521_m1
STAT1	Hs01013996_m1
GAPDH	Hs02786624_g1
FcγRI	Hs00174081_m1
FcγRIIA	Hs01013401_g1
FcγRIIB	Hs00269610_m1

Supplementary Table 5: Taqman qPCR primers. *Assay identifiers for Taqman gene expression assays utilised in this study.*
